# Rare predicted loss of function alleles in Bassoon (BSN) are associated with obesity

**DOI:** 10.1038/s41525-023-00376-7

**Published:** 2023-10-21

**Authors:** Na Zhu, Charles A. LeDuc, Ilene Fennoy, Blandine Laferrère, Claudia A. Doege, Yufeng Shen, Wendy K. Chung, Rudolph L. Leibel

**Affiliations:** 1 Department of Pediatrics, Columbia University Irving Medical Center, New York, NY USA; 2 Department of Systems Biology, Columbia University Irving Medical Center, New York, NY USA; 3 NY Obesity Research Center, Columbia University Irving Medical Center, New York, NY USA; 4 Naomi Berrie Diabetes Center, Columbia University Irving Medical Center, New York, NY USA; 5 Department of Medicine, Columbia University Irving Medical Center, New York, NY USA; 6 Department of Pathology, Columbia University Irving Medical Center, New York, NY 10032 USA; 7 Department of Biomedical Informatics, Columbia University Irving Medical Center, New York, NY USA; 8 JP Sulzberger Columbia Genome Center, Columbia University Irving Medical Center, New York, NY USA; 9 Herbert Irving Comprehensive Cancer Center, Columbia University Irving Medical Center, New York, NY USA

**Keywords:** Metabolic disorders, Medical genomics

## Abstract

Bassoon (*BSN*) is a component of a hetero-dimeric presynaptic cytomatrix protein that orchestrates neurotransmitter release with Piccolo (*PCLO*) from glutamatergic neurons throughout the brain. Heterozygous missense variants in *BSN* have previously been associated with neurodegenerative disorders in humans. We performed an exome-wide association analysis of ultra-rare variants in about 140,000 unrelated individuals from the UK Biobank to search for new genes associated with obesity. We found that rare heterozygous predicted loss of function (pLoF) variants in *BSN* are associated with higher BMI with p-value of 3.6e-12 in the UK biobank cohort. Additionally, we identified two individuals (one of whom has a de novo variant) with a heterozygous pLoF variant in a cohort of early onset or extreme obesity and report the clinical histories of these individuals with non-syndromic obesity with no history of neurobehavioral or cognitive disability. The BMI association was replicated in the All of Us whole genome sequencing data. Heterozygous pLoF *BSN* variants constitute a new etiology for obesity.

## Introduction

By 2030 it is estimated that roughly 50% of adults in the United States will be obese, with 25% having severe obesity^1^. The prevalence of obesity in U.S. adults has increased from 30.5 to 41.9% from 1999 to 2023; the prevalence of severe obesity has increased from 4.7 to 9.2%. Approximately 18% of U.S. children currently are obese^2^. The estimation from twin and GWAS data is that the risk of obesity is 30–50% heritable^3–6^. Changes in underlying genetics cannot be responsible for large increases in the prevalence of obesity over such a short period of time; however, the propensity to gain weight in an environment with ready access to food is largely genetic^7^. Genome-wide association studies have identified many common variants associated with body weight regulation^8–10^. More recently, polygenic risk scores aggregating large numbers genetic variants, each with small contributions to energy homeostasis, can be used to predict obesity deciles in some genetic ancestries^11^. However, the known genome-wide significant loci only explain ~6% of variation in BMI^12,13^. Exome sequencing of large numbers of individuals has accelerated the discovery of rare genetic contributors to quantitative phenotypes such as height^14,15^, celiac disease^16^, and dyslipidemia^17,18^. In many instances the precise mechanistically functional relevance of these associated genetic variants remains unknown.

Recent advances in the treatment of obesity^19^ and hyperlipidemia^20^ have used human genetics to identify genes contributing to extreme phenotypes to understand biology and molecular mechanisms and develop novel interventions. The advent of large-scale exome/genome sequencing in the United Kingdom Biobank (UKBB) and All of Us has extended the ability to assess rare variants at large scale in addition to prior methods of assessing common variants in GWAS. In the current study we combine the power of large population genomic data from the UKBB and All of Us and an extreme obesity cohort recruited at Columbia University. We report the association of predicted loss of function (pLoF) alleles in the gene *BSN* with body mass index (BMI).

## Results

### Cohorts and overview of analysis workflow

We obtained data from three cohorts to identify new obesity risk genes: 1) the UK Biobank (UKBB) study^21,22^ (interim 200k release, Table 1) with exome sequence data and basic phenotype information. We excluded related individuals and individuals with a history of cancer or eating disorders and limited analysis to the 144,496 individuals of European ancestry by principal component analysis. 2) a cohort recruited at Columbia University (the Columbia cohort- summarized in Table 2) enriched in early onset and extreme obesity^23^ with 1598 probands with exome sequencing data, and 3) the All of Us dataset of about 50,000 European ancestry individuals with both BMI and whole genome sequencing data.

**Table 1:** UKBB cohort summary.

BMI (mean, sd) kg/m^2^	27.3, 4.8
age (mean, sd) years	56.5, 8.1
Female: male	110,476: 90,153
With cancer	20,099
With eating disorder	160
European ancestries	1,67,246
Non-European ancestry	33,383
Related samples to be removed	5596

Correlation between age and BMI p = 0.048.

Correlation between age and Sex p = 0.082.

The UKBB cohort used in the analysis. Samples that were coded as having had cancer, eating disorders, or both were removed from the cohort prior to analysis.

Relatedness was estimated using plink King, when sample pairs had a relatedness greater than 0.12 (second degree relative or closer) the sample that had more relatedness to the cohort was excluded. F is female. M is male. EUR indicates European ancestry.

**Table 2:** Patients in Columbia cohort.

	Child-onset	Adult-onset
BMI (mean, sd) kg/m^2^	41.4, 12.4	45.9, 11.9
BMIZ (mean,sd)	6.6, 3.6	
age (mean,sd) years	12.2, 3.5	38.9, 12.3
Female: male	523:377	503:195
European ancestry	322	528
Non-European ancestry	352	170
Total	674	698

Clinical characteristics of the Columbia extreme or early-onset obesity cohort. BMI is body mass index. BMIz is calculated for all children as the BMI z score for gender and age.

**Fig. 1:**
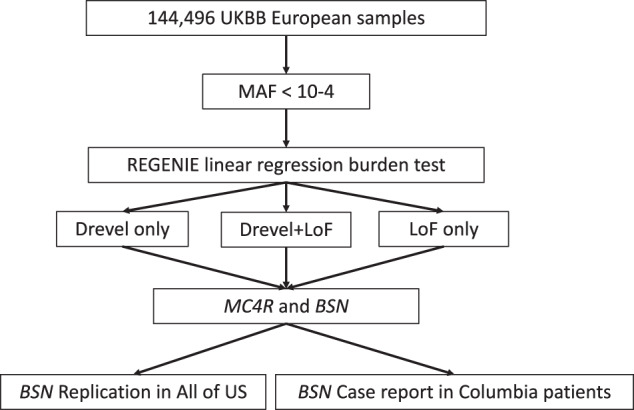
Overall workflow. Only rare (MAF < 10^−4^) likely damaging variants are kept for association analysis. Drevel refers to likely damaging missense variants with REVEL-predicted score above a certain threshold. In the association analysis of the discovery cohort (UKBB), 7 different combinations of variants were tested using the REGENIE linear regression gene-burden test. *BSN* reached significance and was replicated in the All of Us dataset (N ~ 50,000). Two BSN LoF heterozygotes were identified in Columbia patients.

We used the UKBB data as the discovery cohort for association of rare variants and BMI, and then sought additional support in the Columbia cohort and replication in the All of Us data (Fig. 1).

### Association of rare variants with BMI in the discovery cohort

First, we performed an exome-wide scan of risk genes through a linear regression of BMI over counts of rare damaging variants using the UKBB data. For each gene, we tested seven different ways of combining rare (allele frequency < 1e-4 in the population) variants, including protein truncating variants (inclusion or not), and predicted damaging missense variants by REVEL at variable thresholds. We carried out the association test using REGENIE^24^, with age, Townsend deprivation index at recruitment, smoking /alcohol status, sex, the first eight principal components, and genetic heterozygosity as covariates (Q-Q plot for UKBB in Supplementary Figure 1). We identified two genes associated with BMI with exome-wide significance (Table 3, one is *MC4R* (beta = 1.4, *p*-value = 5.0e-10, genomic position for *MC4R* variants in Supplementary Fig. 2, BMI distribution in Supplementary figure 3), a known obesity gene; the other is *BSN* (beta=6.2, *p* value = 3.6e-12), a novel putative obesity risk gene (individual variants listed in Supplementary Tables 1 (*MC4R*) and 2(*BSN*)).

**Table 3:** REGENIE linear regression for UKBB only.

Gene	GeneDescription	MASK	A1FREQ	BETA	SE	CHISQ	*p*-value
*BSN*	bassoon presynaptic cytomatrix protein	LoF+Drevel > =0.75	9.34E-05	6.21	0.89	48.29	3.60E-12
*MC4R*	melanocortin 4 receptor	LoF+Drevel > =0.25	1.47E-03	1.40	0.23	38.66	5.00E-10
*PCSK1*	proprotein convertase subtilisin/kexin type 1	LoF+Drevel > =0.75	9.34E-04	1.42	0.28	25.17	5.20E-07
*PHF3*	PHD finger protein 3	LoF+Drevel > =0.75	1.56E-04	3.26	0.69	22.18	2.48E-06
*ALOX5*	arachidonate 5-lipoxygenase	Drevel > =0.5	1.56E-03	1.00	0.22	20.95	4.71E-06
*NCR2*	natural cytotoxicity triggering receptor 2	Drevel > =0.25	7.96E-05	4.29	0.97	19.63	9.41E-06

UKBB REGENIE linear regression result.

The BMI association results for UKBB cohort using REGENIE. Drevel refers to likely damaging missense variants with REVEL-predicted score about a certain threshold. Genes with *p* value < e-5 are listed with the smallest *p* value for each gene listed. The *p* value significance threshold after Bonferroni correction was 3.57e-7. *BSN* was a novel gene that reached genome wide significance. *MC4R* and *PCSK1* are known obesity risk genes.

**Fig. 2:**
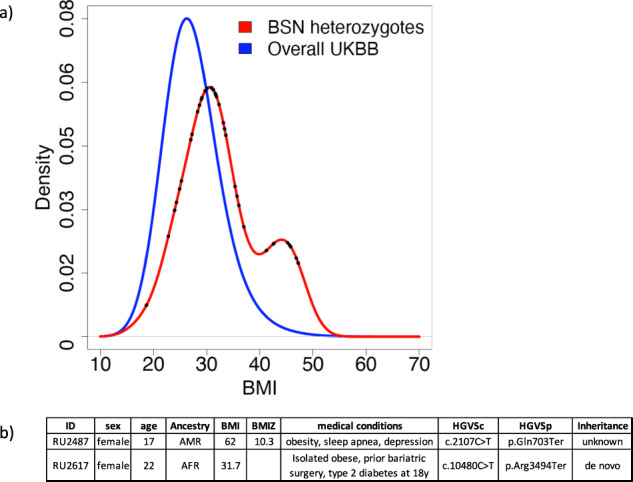
BMI density distribution. **a** The BMI density distribution for pLoF *BSN* heterozygotes is shifted to a higher BMI than the overall UKBB cohort. A.) There is an apparent bi-modal distribution for the *BSN* pLoF heterozygotes. This distribution is not explained by putative protein functional consequences (CADD score), genomic physical location, phylogenetic conservation, epigenetic markers, or histone modification across different stages. The bimodal may be simply due to the small number of carriers and the bin size, if the bin size is increased from 2 to 5, the binomial distribution goes away. The distribution difference between overall cohort and UKBB heterozygotes was tested using the Kolmogorow-Smirnov method (*p* = 1.4e-05). The dots in the red curve represent the BMI of the individuals with predicted deleterious *BSN* variants. **b** Phenotype of *BSN* pLoF heterozygotes in the Columbia cohort.

The association of *BSN* with BMI is primarily driven by protein-truncating variants and damaging missense variants with REVEL > 0.75. Twenty-seven individuals have heterozygous genotypes for one of these variants (aggregated allele frequency = 9.3e-5). Figure 2a shows the BMI distribution of *BSN* predicted deleterious heterozygotes compared to the overall UKBB population (Kolmogorov-Smirnov *p*-value = 1.4e-05).

### BSN pLOF carriers from the Columbia cohort

We identified two heterozygous pLoF *BSN* alleles in the Columbia cohort (Fig. 2b). RU2487 is heterozygous for a de novo p.Gln703X allele in *BSN*. At the time of the last assessment, she was a 23-year-old Latina woman with a history of severe obesity and type 2 diabetes mellitus diagnosed at age 19 years at which time her HbA1c was 7.4%. She was amenorrheic and had extensive acanthosis nigricans, dyslipidemia, hypothyroidism, and hyperandrogenism. Her maximal weight was 113 kg at age 20. She had gastric bypass surgery for weight loss at age 21. Immediately prior to bariatric surgery, her BMI was 39.7 kg/m^2^. Her oral glucose tolerance test prior to bariatric surgery showed euglycemic hyperinsulinemia. Her nadir body weight after surgery was 77 kg; 2 years post-operatively she weighed 101 kg. She reports frequently feeling very hungry. She is a college graduate with no academic or cognitive difficulties nor history of psychiatric diagnoses. She has no family history of obesity or type 2 diabetes.

RU2617 is an African American female heterozygous for a p.R3494X variant in *BSN*; the allele was not inherited from the only parent available for genetic analysis. At the time of her initial evaluation, the patient was 15.7 years old with body weight of 162 kg and height of 160.9 cm (BMI = 62.6 kg/m^2^). Her waist circumference was 158 cm. She was 11 years old at menarche and had no history of irregular periods. She had obstructive sleep apnea requiring continuous positive airway pressure. She initially had a normal glucose tolerance test with normal fasting glucose and HbA1c = 6.3%; however, she subsequently developed impaired fasting blood glucose of 105 mg/dl with persistently elevated HbA1c. She had laparoscopic adjustable gastric banding at 17.5 years. At 3 years post operatively, her weight had declined to 134.2 kg and her height had increased to 163 cm (BMI of 50.5 kg/m^2^). HbA1c normalized to 5.2%.

### Association of BMI-correlated traits in BSN

The association between *BSN* and the traits correlated with BMI tested using REGENIE (Supplementary Table 3) showed arm, leg and trunk fat mass and leg fat-free mass and leg predicted mass reached genome-wide significance. We also tested the association between *BSN* and ICD10 diagnoses (Supplementary Table 4) using the binomial test. No diagnosis was significantly associated with *BSN* after correction for multiple testing.

### Replication analysis using All of Us data

We sought to replicate the association of *BSN* using the All of Us cohort. As of February 2023, there were 98,622 subjects for whom both whole genome sequencing and clinical data are available. Half of the participants (47,897) are unrelated and of European ancestry. For each participant, we used the highest recorded BMI, giving a cohort average BMI of 30.1 +/− 7.8 kg/m^2^. In the cohort, 12 European individuals were heterozygous for *BSN* pLoF variants, with an average BMI of 37.0 +/− 5.7 kg/m^2^. Using sex, age, income, and deprivation index as covariates, we tested the association between BMI and *BSN* genotype using linear regression and found a significant association (*p* value = 0.0075) with beta=6.3, a large effect size similar to the estimation from the UK Biobank cohort. Additionally, we identified six *BSN* pLoF heterozygotes among the non-European participants (mean BMI 31.5 (SD = 8.5 kg/m^2^); BMI range = 22–45; 3/6 with BMI > 30.0; Supplementary Table 5). Thus, the *BSN* obesity association observed in the UKBB and Columbia cohorts was replicated in the All of Us cohort.

## Discussion

We have identified a gene, *BSN*, for which we have demonstrated an association of rare pLoF variants with obesity in two independent large cohorts: the UKBB and All of Us, with similarly large effect size. Additionally, we identified extremely obese individuals in the Columbia cohort of early onset and/or extreme obesity, including an individual with extreme, early onset obesity associated with a de novo pLoF allele. There is no evidence that these variants are associated with intellectual disability or cognitive impairment, including direct assessment of two individuals in the Columbia cohort.

*BSN* (bassoon) is expressed primarily in the brain (including embryonic and adult brain regions that impact feeding behavior^25^), inner hair cell ribbons, and the retina of mammals. Bassoon is a presynaptic scaffold protein localized in the cytomatrix at the active zone (CAZ) where it functions to orchestrate neurotransmitter release. Bassoon participates in the formation of Golgi-derived Piccolo-Bassoon transport vesicles that are axonally transported to newly formed synaptic contacts. Bassoon is associated with activity-dependent short- and long-term neuronal plasticity^26^.

Bassoon is expressed during early neuronal differentiation, is selectively sorted into axons and is among the first proteins to arrive at nascent synapses^26^. The release of neurotransmitters from the presynaptic terminal involves the active zone (AZ). The AZ includes an electron-dense protein meshwork, the presynaptic cytomatrix. Bassoon is one of several scaffolding proteins (along with Piccolo (*PCLO*), *RIM*, *MUNC13*, and *ELKS*) within the presynaptic cytomatrix. *BSN* and *PCLO* are structurally related, interact, and are the largest active-zone-specific proteins. Unlike other the proteins in the AZ that are evolutionally conserved down to C. elegans, Piccolo and Bassoon are only found in vertebrates^27^.

Mice homozygous for LoF *Bsn* alleles have reduced synaptic transmission that is primarily caused by the inactivation of a significant fraction of glutamatergic synapses. These mice have spontaneous epileptic seizures. Bassoon is not essential for synapse formation but is essential for regulated neurotransmitter release from a subset of glutamatergic synapses^28^. At the ultrastructural level, these inactive synapses cannot be distinguished from functional synapses. These homozygous Bassoon mutant mice have seizures with structural brain alterations including enlarged hippocampi and cerebral cortices^29^. These animals are not obese.

Bassoon is involved in the maintenance of the integrity of AZ^30^. Glutamatergic synapses from *Bsn* knockout mice exhibit enhanced short-term synaptic depression with a high percentage of silent synapses but have no gross structural defects^31^, presumably due to the significant functional redundancy with Picolo. When both proteins are absent from glutamatergic synapses, the cells undergo synapse degeneration^32^.

*BSN* was originally identified while attempting to identify expressed cerebellar transcripts in patients with multiple system atrophy, a rare progressive neurodegenerative disease characterized by cerebellar symptoms, parkinsonism, and autonomic dysfunction^33^. This study did not find coding mutations in *BSN* but first identified *BSN* as a new transcript that could be cloned from the cerebellum of these patients. *BSN* acts in concert with Parkin RBR E3 Ubiquitin Protein Ligase (PRKN) to control presynaptic autophagy and maintain homeostatic presynaptic proteostasis and synaptic vesicle turnover^34^. Human heterozygous missense variants in *BSN* have been implicated in neurodevelopmental and neurodegenerative disorders including progressive supranuclear palsy-like syndrome, a rare neurodegenerative tauopathy^35^.

We have implicated heterozygous pLoF variants in *BSN* as a new genetic etiology for human obesity that is not associated with adverse impact on cognition or other neurobehavioral phenotypes. The expression of *BSN* throughout the brain suggests that gene dosage could contribute to hyperphagia through both homeostatic and hedonic circuits^36^. Additional detailed phenotypic assessment – ideally of individuals prior to the onset of obesity – will be required to assess this point. *BSN* is expressed in the synapses of glutamatergic neurons and hypothalamic neurons mechanistically tied to ingestive behaviors^31,37–39^. The valence of these effects is consistent with hyperphagic obesity conveyed by hypomorphic alleles.

## Methods

We ran REGENIE for rare variants association in UKBB data to detect the risk genes associated with BMI, and then sought additional support in the Columbia cohort and replication in the All of Us data (Fig. 1). Informed consent was obtained from all human participants.

### UKBB cohort

For this analysis, we included 200,643 individuals from the UK biobank^22^. The average age of this cohort is 56.4 +/− 8.1 years; mean BMI of 27.3 +/− 4.8 kg/m^2^; 55.1% female (Table 1).

### Columbia cohort

The Columbia University early onset and/or extreme obesity cohort was collected using protocols approved by the Institutional Review Boards at Columbia University Irving Medical Center (New York, NY) and The Rockefeller University (New York, NY). The cohort consists of 1598 individuals from 903 families. Obesity was defined as described below. Of the 903 families, 122 constitute affected child/parent trios. The remaining 781 families have 1372 affected (890 females and 482 males) and 226 unaffected family members. Cohort details have been described previously^23,40^. Approximately half of the probands were pediatric (either at time of recruitment or obesity onset age younger than 19 years old with 674 participants having a BMI Z score >=2; average age at enrollment 6.6 + /- 3.6 years) and half adults (obesity onset or recruitment age at least 19 years old with 698 adults with BMI > = 30 kg/m^2^; average age 51.5 + /- 12.0 years) (Table 2). Samples were exome sequenced using xGen and SeqCap VCRome Capture. Greater than 99% of samples had depth of coverage > 10 in 80% of target regions.

### All of Us data

The current release (June 2022) of the All of Us data includes whole genome sequencing for 98,622 individuals (58,190 females and 38,290 males). The average age of this cohort is 52.6 +/− 16.9 years; mean BMI is 30.9 +/− 9.0 kg/m^2^.

#### Bioinformatic analysis of exome or genome sequencing data

##### Columbia cohort

Paired-end reads were mapped and aligned to the human reference genome (version GRCh38/hg38, accession GCA 000001405.15) using BWA-MEM^41^. Picard v1.93 MarkDuplicates (http://broadinstitute.github.io/picard/) was used to identify and flag PCR duplicates and GATK v4.1 HaplotypeCaller^42^ in Reference Confidence Model mode to generate individual-level gVCF files from the aligned sequence data. We performed joint calling of variants from the obesity cohorts using GATK variant caller.

##### Ancestry prediction and relatedness check

We predicted the ancestry and relatedness in the Columbia cohort using Peddy^43^. Relatedness prediction in the UKBB samples, due to the large sample size, was completed with plink King^44^. To ensure that private mutations carried in individual families were not over-counted, samples with a second-degree relationship or closer (a kinship coefficient greater than 0.12 in King or 0.25 in Peddy) had the relative who was more related to the overall cohort excluded.

##### Variant annotation

We used the Ensembl Variant Effect Predictor (VEP, Ensemble 93)^45^ to annotate variant function and ANNOVAR^46^ to aggregate variant population frequencies and for in silico predictions of deleteriousness. Rare variants were defined by a population frequency < 10^−4^ in gnomAD WES and WGS^47^. Deleterious variants were defined as predicted loss-of-function (pLoF: including premature stop-gain, stop-loss, frameshift indels, canonical splicing variants and multi-exon deletions) or predicted damaging missense (Dmis) based on REVEL^48^ score thresholds. The same annotation pipeline was used for Columbia, UKBB, and All of US variant annotation.

#### Statistical analysis

##### UKBB cohort

After excluding related individuals and individuals with a history of cancer or eating disorder, 144,496 unrelated European individuals were selected for quantitative trait (BMI) association analysis^21,47^. We collapsed rare variants based on allele frequency and predicted variant deleteriousness. The variants were partitioned into cohort frequency <10^−4^ as well as 7 variant functional groups. The variant functional groups were missense variants with REVEL >=x, where x is 0.25, 0.5, 0.75, with or without pLoF variants (7 combinations). Genes having less than 15 heterozygotes in a test group were removed. The significance threshold was set to 3.5e-07 ( = 0.05/ (7*20,000)). We then tested the quantitative BMI for the 144 K UKBB individuals using REGENIE^24^, which accounts for relatedness, population structure and polygenicity. We included age, Townsend deprivation index at recruitment, smoking /alcohol status, sex, the first 8 principal components, and genetic heterozygosity as covariates. REGENIE resolved the gene-based association tests in the large UKBB dataset with no inflation or deflation in the synonymous variants with the gene-based tests (Sup Fig. 1a). The type I error rate was well controlled for pLoF and Dmis variants in gene-based tests, showing minor inflation in the QQ plot (Sup Fig. 1b).

##### Columbia cohort

When there were multiple individuals with obesity in a family, the most severely affected was defined as the proband (either the child with the highest Z-score; or the adult with the highest BMI if there were only adults in the family).

We defined the threshold for genome-wide significance by Bonferroni correction for multiple testing (*n* = 20,000*7, threshold *p*-value = 3.57e-7) (workflow shown in Fig. 2).

##### All of Us

To attempt to replicate findings from the UKBB analysis and Columbia Cohort, we ran linear regression on the 48,722 European ancestry individuals from the All of Us dataset using their provided cloud-based research platform to test the association between BMI and *BSN* deleterious variants using age, sex, deprivation index and median income as covariates.

All analyses were under the auspices of the Columbia University IRB “Molecular Genetic Analysis of Obesity and Non-Insulin Dependent Diabetes Mellitus” IRB #: AAAA4485. All participants provided written or electronic informed consent to take part in their respective study (Columbia, UK Biobank, or All of Us).

### Reporting summary

Further information on research design is available in the Nature Research Reporting Summary linked to this article.

### Supplementary information

Supplementary figures and tables

REPORTING SUMMARY

## Data Availability

UKBB genotypic and phenotypic data are available to approved investigators via the UK Biobank study (www.ukbiobank.ac.uk/). Additional information about registration for access to the data is available at www.ukbiobank.ac.uk/register-apply/. Data access for approved applications requires a data transfer agreement between the researcher’s institution and UK Biobank, the terms of which are available on the UK Biobank website (www.ukbiobank.ac.uk/media/ezrderzw/applicant-mta.pdf). Original All of Us Biobank data are available to registered and approved All of Us researchers (https://www.researchallofus.org/register/). Genetic data requires controlled tier access, which researchers can register for through their institutions.

## Code availability

We used REGENIE for statistical analysis. The software is written by a team in Regeneron, available on GitHub: https://nam02.safelinks.protection.outlook.com/?url=https%3A%2F%2Frgcgithub.github.io%2Fregenie%2F&data=05%7C01%7Cnz2274%40cumc.columbia.edu%7Cb05e1e0311ea4ad5915308db8868e3e7%7Cb0002a9b0017404d97dc3d3bab09be81%7C0%7C0%7C638253756299911043%7CUnknown%7CTWFpbGZsb3d8eyJWIjoiMC4wLjAwMDAiLCJQIjoiV2luMzIiLCJBTiI6Ik1haWwiLCJXVCI6Mn0%3D%7C3000%7C%7C%7C&sdata=IOOvWWxc9Qpsd2pqK9p9k1oRRMPWoDnbjqYdYhtFYUw%3D&reserved=0.
