# Supplementary material for: Rare predicted loss of function alleles in Bassoon (BSN) are associated with obesity
Source: NPJ Genom Med. 2023 Oct 21;8:33. doi: 10.1038/s41525-023-00376-7 (PMC10590409; doi:10.1038/s41525-023-00376-7)
Supplement: Supplementary file 1 — Supplementary figures and tables [file 41525_2023_376_MOESM1_ESM.pdf]

Supplementary Figures:

Supplementary Figure 1.

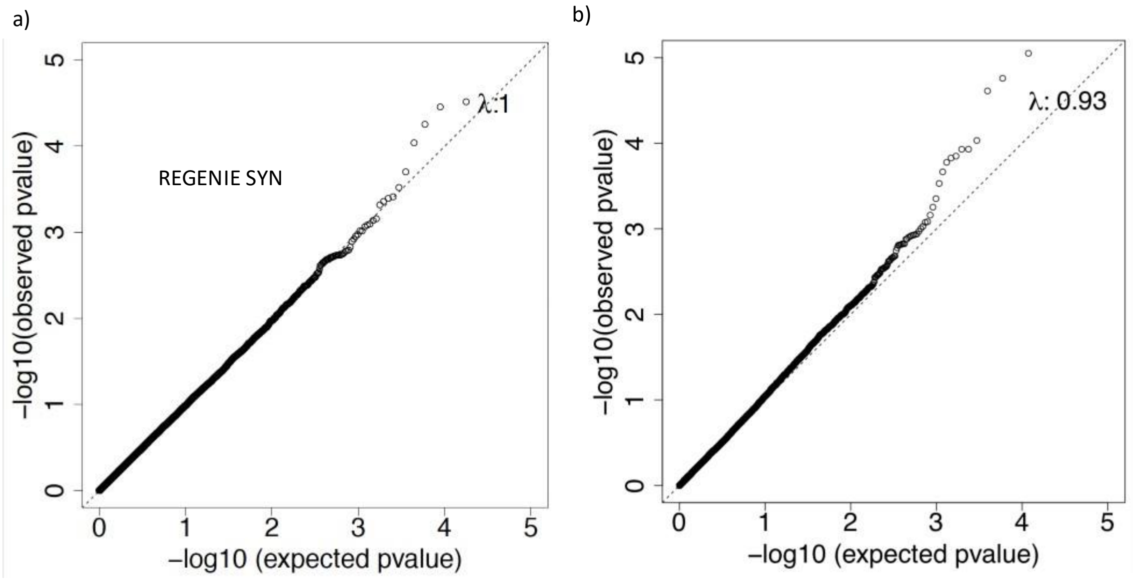

Supplementary Figure 2

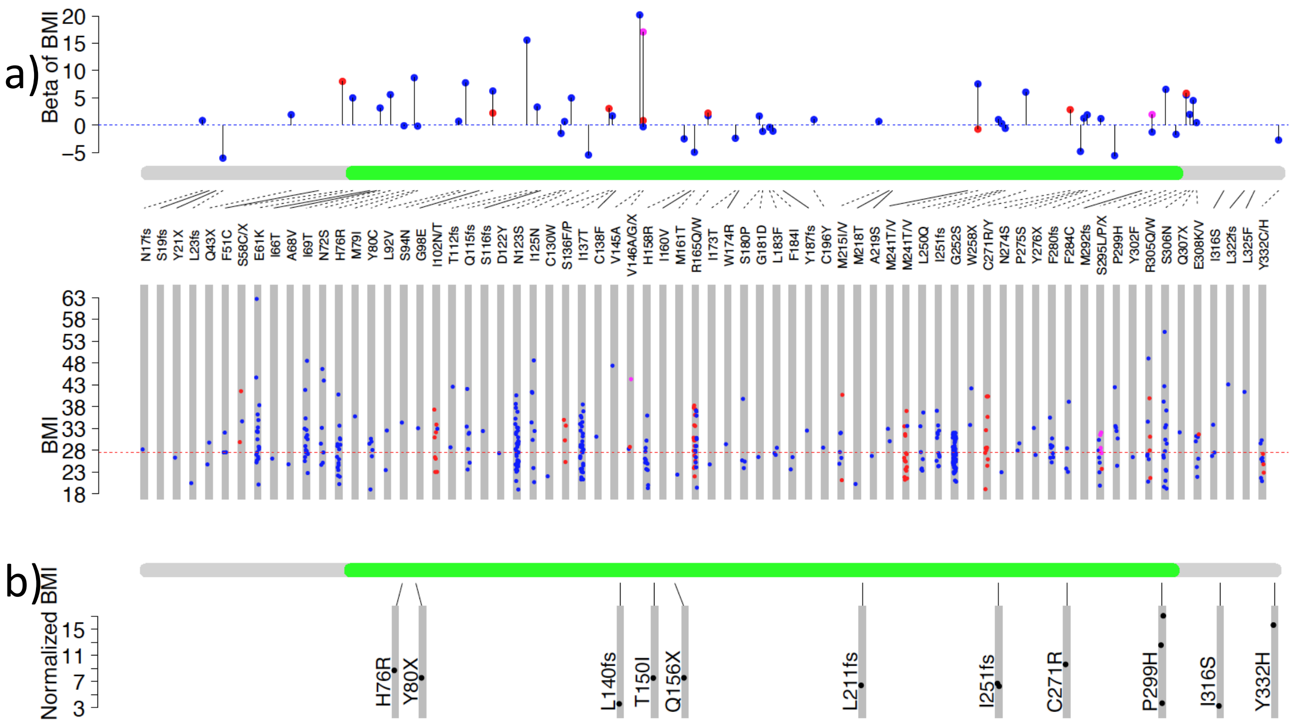

Supplementary Figure 3

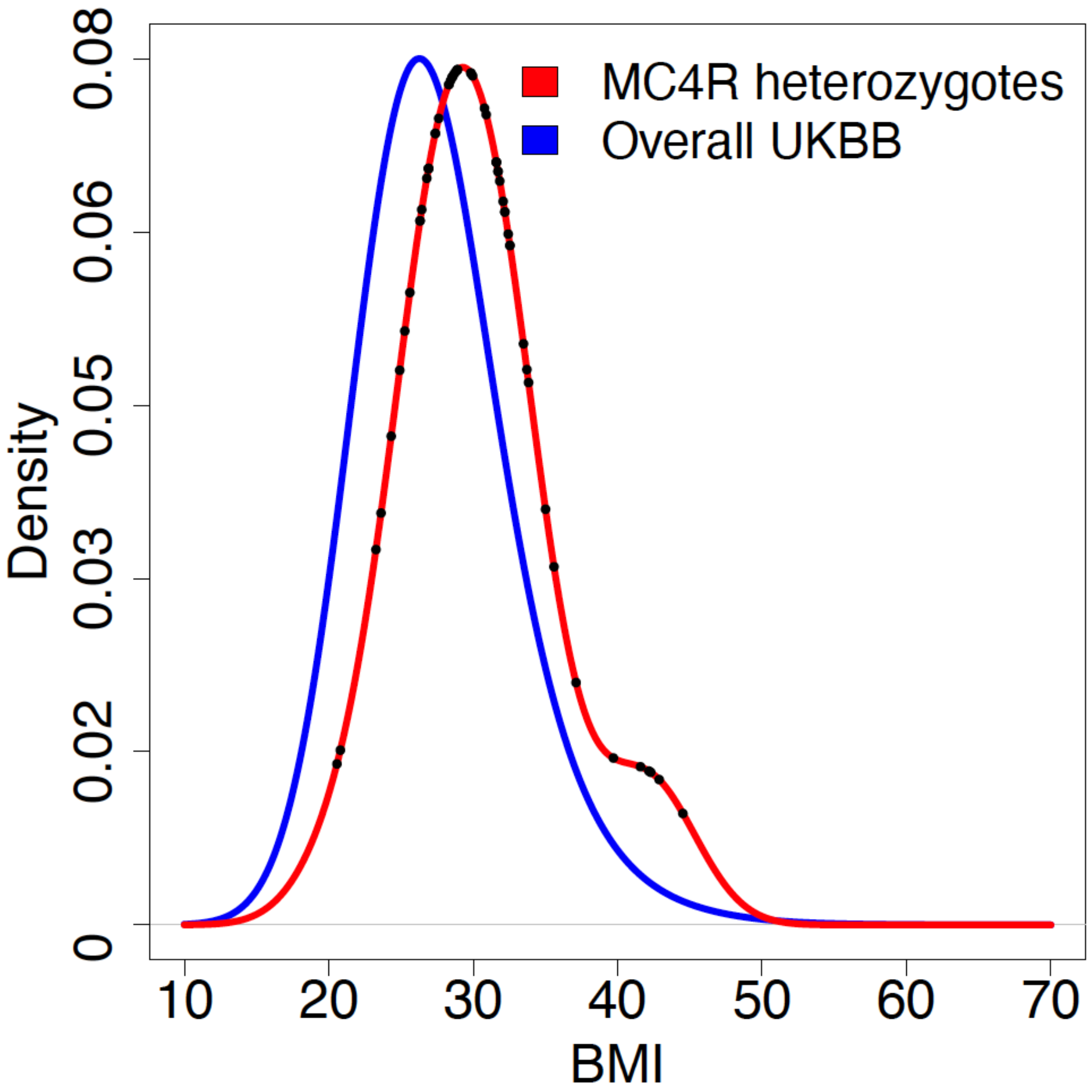

Supplementary tables:

Supplementary table 1

Deleterious MC4R variants, transcript ID:ENST00000299766, in UKBB cohort											
proband	BMI	Sex	age	HGVSp	Consequence	UKBB MAF	CADD phred	REVEL score	TOPMed AF	gnomAD3 AF	gnomAD AF
3093062	28.2	female	61	p.Asn17GlufsTer13	frameshift_variant	2.5E-06	.	.	8.0E-06	7.0E-06	4.0E-06
4704264	NA	NA	NA	p.Ser19AlafsTer34	frameshift_variant	2.5E-06	.	.	.	.	4.0E-06
5671748	26.3	female	53	p.Tyr21Ter	stop_gained	2.5E-06	.	.	.	.	1.2E-05
1177906	20.5	female	68	p.Leu23ArgfsTer30	frameshift_variant	2.5E-06	.	.	.	.	.
2106752	24.8	female	62	p.Gln43Ter	stop_gained	5.0E-06	40	.	.	.	4.0E-06
5331630	29.8	male	68	p.Gln43Ter	stop_gained	5.0E-06	40	.	.	.	4.0E-06
2441968	27.5	male	64	p.Phe51Cys	missense_variant	7.5E-06	27.8	0.66	.	.	.
3630335	32.0	female	65	p.Phe51Cys	missense_variant	7.5E-06	27.8	0.66	.	.	.
3952605	27.5	male	68	p.Phe51Cys	missense_variant	7.5E-06	27.8	0.66	.	.	.
1028732	29.9	female	64	p.Ser58AlafsTer7	frameshift_variant	5.0E-06	.	.	.	.	4.0E-06
5298968	41.5	female	55	p.Ser58AlafsTer7	frameshift_variant	5.0E-06	.	.	.	.	4.0E-06
1843997	34.6	female	52	p.Ser58Cys	missense_variant	2.5E-06	26.7	0.61	.	.	.
1076923	NA	NA	NA	p.Glu61Lys	missense_variant	5.5E-05	29.6	0.75	2.4E-05	1.4E-05	2.0E-05
1218226	44.7	female	60	p.Glu61Lys	missense_variant	5.5E-05	29.6	0.75	2.4E-05	1.4E-05	2.0E-05
1613267	27.0	male	65	p.Glu61Lys	missense_variant	5.5E-05	29.6	0.75	2.4E-05	1.4E-05	2.0E-05
1769858	25.2	female	49	p.Glu61Lys	missense_variant	5.5E-05	29.6	0.75	2.4E-05	1.4E-05	2.0E-05
2059112	62.7	female	59	p.Glu61Lys	missense_variant	5.5E-05	29.6	0.75	2.4E-05	1.4E-05	2.0E-05
2177681	25.6	female	48	p.Glu61Lys	missense_variant	5.5E-05	29.6	0.75	2.4E-05	1.4E-05	2.0E-05
2214923	32.3	female	65	p.Glu61Lys	missense_variant	5.5E-05	29.6	0.75	2.4E-05	1.4E-05	2.0E-05
2327276	27.5	female	67	p.Glu61Lys	missense_variant	5.5E-05	29.6	0.75	2.4E-05	1.4E-05	2.0E-05
2370118	32.4	male	64	p.Glu61Lys	missense_variant	5.5E-05	29.6	0.75	2.4E-05	1.4E-05	2.0E-05
2413810	30.5	female	46	p.Glu61Lys	missense_variant	5.5E-05	29.6	0.75	2.4E-05	1.4E-05	2.0E-05
2413908	36.2	male	51	p.Glu61Lys	missense_variant	5.5E-05	29.6	0.75	2.4E-05	1.4E-05	2.0E-05
2567264	32.2	male	44	p.Glu61Lys	missense_variant	5.5E-05	29.6	0.75	2.4E-05	1.4E-05	2.0E-05

2598583	33.2	female	69	p.Glu61Lys	missense_variant	5.5E-05	29.6	0.75	2.4E-05	1.4E-05	2.0E-05
3174169	26.5	female	57	p.Glu61Lys	missense_variant	5.5E-05	29.6	0.75	2.4E-05	1.4E-05	2.0E-05
3475408	26.1	female	44	p.Glu61Lys	missense_variant	5.5E-05	29.6	0.75	2.4E-05	1.4E-05	2.0E-05
3668972	34.9	male	51	p.Glu61Lys	missense_variant	5.5E-05	29.6	0.75	2.4E-05	1.4E-05	2.0E-05
3700423	20.2	female	49	p.Glu61Lys	missense_variant	5.5E-05	29.6	0.75	2.4E-05	1.4E-05	2.0E-05
4195426	28.8	female	57	p.Glu61Lys	missense_variant	5.5E-05	29.6	0.75	2.4E-05	1.4E-05	2.0E-05
4492964	NA	NA	NA	p.Glu61Lys	missense_variant	5.5E-05	29.6	0.75	2.4E-05	1.4E-05	2.0E-05
5322703	28.2	male	62	p.Glu61Lys	missense_variant	5.5E-05	29.6	0.75	2.4E-05	1.4E-05	2.0E-05
5510811	28.7	male	51	p.Glu61Lys	missense_variant	5.5E-05	29.6	0.75	2.4E-05	1.4E-05	2.0E-05
5673198	38.3	female	57	p.Glu61Lys	missense_variant	5.5E-05	29.6	0.75	2.4E-05	1.4E-05	2.0E-05
2191173	26.1	female	66	p.Ile66Thr	missense_variant	2.5E-06	29.7	0.82	.	.	.
1094515	24.8	female	42	p.Ala68Val	missense_variant	2.5E-06	27.9	0.51	.	.	4.0E-06
1384458	33.0	female	56	p.Ile69Thr	missense_variant	4.7E-05	29.9	0.59	.	.	4.0E-06
1499128	25.5	female	64	p.Ile69Thr	missense_variant	4.7E-05	29.9	0.59	.	.	4.0E-06
1726939	26.4	male	66	p.Ile69Thr	missense_variant	4.7E-05	29.9	0.59	.	.	4.0E-06
2244512	41.8	female	65	p.Ile69Thr	missense_variant	4.7E-05	29.9	0.59	.	.	4.0E-06
2288389	29.0	female	60	p.Ile69Thr	missense_variant	4.7E-05	29.9	0.59	.	.	4.0E-06
2386704	35.3	female	59	p.Ile69Thr	missense_variant	4.7E-05	29.9	0.59	.	.	4.0E-06
2561183	28.1	male	60	p.Ile69Thr	missense_variant	4.7E-05	29.9	0.59	.	.	4.0E-06
2633487	27.6	female	58	p.Ile69Thr	missense_variant	4.7E-05	29.9	0.59	.	.	4.0E-06
3402124	31.0	male	56	p.Ile69Thr	missense_variant	4.7E-05	29.9	0.59	.	.	4.0E-06
3524352	27.5	male	64	p.Ile69Thr	missense_variant	4.7E-05	29.9	0.59	.	.	4.0E-06
3538424	32.7	female	46	p.Ile69Thr	missense_variant	4.7E-05	29.9	0.59	.	.	4.0E-06
4212719	27.8	female	67	p.Ile69Thr	missense_variant	4.7E-05	29.9	0.59	.	.	4.0E-06
4303560	NA	NA	NA	p.Ile69Thr	missense_variant	4.7E-05	29.9	0.59	.	.	4.0E-06
4947078	48.5	male	59	p.Ile69Thr	missense_variant	4.7E-05	29.9	0.59	.	.	4.0E-06
5415758	31.5	female	65	p.Ile69Thr	missense_variant	4.7E-05	29.9	0.59	.	.	4.0E-06
5672319	NA	NA	NA	p.Ile69Thr	missense_variant	4.7E-05	29.9	0.59	.	.	4.0E-06

5850314	30.5	female	55	p.Ile69Thr	missense_variant	4.7E-05	29.9	0.59	.	.	4.0E-06
5993331	22.8	male	68	p.Ile69Thr	missense_variant	4.7E-05	29.9	0.59	.	.	4.0E-06
5994148	27.2	female	60	p.Ile69Thr	missense_variant	4.7E-05	29.9	0.59	.	.	4.0E-06
2824673	29.5	male	59	p.Asn72Ser	missense_variant	1.7E-05	25.4	0.42	.	7.0E-06	8.0E-06
4613880	24.7	female	58	p.Asn72Ser	missense_variant	1.7E-05	25.4	0.42	.	7.0E-06	8.0E-06
4621310	33.1	female	58	p.Asn72Ser	missense_variant	1.7E-05	25.4	0.42	.	7.0E-06	8.0E-06
4972022	46.6	female	55	p.Asn72Ser	missense_variant	1.7E-05	25.4	0.42	.	7.0E-06	8.0E-06
5150061	25.1	female	62	p.Asn72Ser	missense_variant	1.7E-05	25.4	0.42	.	7.0E-06	8.0E-06
5415010	27.5	female	62	p.Asn72Ser	missense_variant	1.7E-05	25.4	0.42	.	7.0E-06	8.0E-06
5758971	44.0	female	52	p.Asn72Ser	missense_variant	1.7E-05	25.4	0.42	.	7.0E-06	8.0E-06
1683286	26.5	male	42	p.His76Arg	missense_variant	5.7E-05	25.8	0.71	4.0E-05	7.0E-05	2.0E-05
1841134	NA	NA	NA	p.His76Arg	missense_variant	5.7E-05	25.8	0.71	4.0E-05	7.0E-05	2.0E-05
1969867	28.9	female	60	p.His76Arg	missense_variant	5.7E-05	25.8	0.71	4.0E-05	7.0E-05	2.0E-05
2173541	29.4	female	51	p.His76Arg	missense_variant	5.7E-05	25.8	0.71	4.0E-05	7.0E-05	2.0E-05
3156505	24.1	female	44	p.His76Arg	missense_variant	5.7E-05	25.8	0.71	4.0E-05	7.0E-05	2.0E-05
3326785	29.0	male	55	p.His76Arg	missense_variant	5.7E-05	25.8	0.71	4.0E-05	7.0E-05	2.0E-05
3348406	22.2	male	41	p.His76Arg	missense_variant	5.7E-05	25.8	0.71	4.0E-05	7.0E-05	2.0E-05
3645221	NA	NA	NA	p.His76Arg	missense_variant	5.7E-05	25.8	0.71	4.0E-05	7.0E-05	2.0E-05
3846756	23.5	female	43	p.His76Arg	missense_variant	5.7E-05	25.8	0.71	4.0E-05	7.0E-05	2.0E-05
3881159	40.8	female	62	p.His76Arg	missense_variant	5.7E-05	25.8	0.71	4.0E-05	7.0E-05	2.0E-05
3908576	25.1	male	69	p.His76Arg	missense_variant	5.7E-05	25.8	0.71	4.0E-05	7.0E-05	2.0E-05
3922382	24.6	female	68	p.His76Arg	missense_variant	5.7E-05	25.8	0.71	4.0E-05	7.0E-05	2.0E-05
3961449	NA	male	69	p.His76Arg	missense_variant	5.7E-05	25.8	0.71	4.0E-05	7.0E-05	2.0E-05
4294073	25.9	male	55	p.His76Arg	missense_variant	5.7E-05	25.8	0.71	4.0E-05	7.0E-05	2.0E-05
4716868	28.2	female	60	p.His76Arg	missense_variant	5.7E-05	25.8	0.71	4.0E-05	7.0E-05	2.0E-05
4739507	20.3	female	62	p.His76Arg	missense_variant	5.7E-05	25.8	0.71	4.0E-05	7.0E-05	2.0E-05
4964816	33.6	female	55	p.His76Arg	missense_variant	5.7E-05	25.8	0.71	4.0E-05	7.0E-05	2.0E-05
5205380	NA	NA	NA	p.His76Arg	missense_variant	5.7E-05	25.8	0.71	4.0E-05	7.0E-05	2.0E-05

5314657	22.0	male	59	p.His76Arg	missense_variant	5.7E-05	25.8	0.71	4.0E-05	7.0E-05	2.0E-05
5324120	30.7	male	59	p.His76Arg	missense_variant	5.7E-05	25.8	0.71	4.0E-05	7.0E-05	2.0E-05
5697734	NA	NA	NA	p.His76Arg	missense_variant	5.7E-05	25.8	0.71	4.0E-05	7.0E-05	2.0E-05
5745263	30.5	male	59	p.His76Arg	missense_variant	5.7E-05	25.8	0.71	4.0E-05	7.0E-05	2.0E-05
5886872	29.3	female	58	p.His76Arg	missense_variant	5.7E-05	25.8	0.71	4.0E-05	7.0E-05	2.0E-05
2679542	NA	NA	NA	p.Met79Ile	missense_variant	5.0E-06	25	0.61	.	.	4.0E-06
4237573	35.8	female	59	p.Met79Ile	missense_variant	5.0E-06	25	0.61	.	.	4.0E-06
1441982	29.5	female	48	p.Tyr80Cys	missense_variant	1.7E-05	28.5	0.72	.	7.0E-06	.
2379973	NA	NA	NA	p.Tyr80Cys	missense_variant	1.7E-05	28.5	0.72	.	7.0E-06	.
3268201	19.0	female	40	p.Tyr80Cys	missense_variant	1.7E-05	28.5	0.72	.	7.0E-06	.
3584926	30.7	male	61	p.Tyr80Cys	missense_variant	1.7E-05	28.5	0.72	.	7.0E-06	.
4507572	30.0	female	63	p.Tyr80Cys	missense_variant	1.7E-05	28.5	0.72	.	7.0E-06	.
4519704	26.6	male	47	p.Tyr80Cys	missense_variant	1.7E-05	28.5	0.72	.	7.0E-06	.
4649042	28.1	male	60	p.Tyr80Cys	missense_variant	1.7E-05	28.5	0.72	.	7.0E-06	.
1664363	23.5	male	67	p.Leu92Val	missense_variant	7.5E-06	23.9	0.38	.	.	.
2779040	32.5	female	58	p.Leu92Val	missense_variant	7.5E-06	23.9	0.38	.	.	.
5158191	NA	male	41	p.Leu92Val	missense_variant	7.5E-06	23.9	0.38	.	.	.
1019170	34.3	female	64	p.Ser94Asn	missense_variant	2.5E-06	27.3	0.52	.	.	8.0E-06
2051276	33.1	male	51	p.Gly98Glu	missense_variant	2.5E-06	24	0.39	.	.	.
1325432	37.3	male	63	p.Ile102Thr	missense_variant	2.2E-05	26.1	0.56	.	.	4.0E-06
1746820	31.0	male	61	p.Ile102Thr	missense_variant	2.2E-05	26.1	0.56	.	.	4.0E-06
1815760	26.4	female	61	p.Ile102Thr	missense_variant	2.2E-05	26.1	0.56	.	.	4.0E-06
2318512	23.0	female	47	p.Ile102Thr	missense_variant	2.2E-05	26.1	0.56	.	.	4.0E-06
3777092	26.1	male	60	p.Ile102Thr	missense_variant	2.2E-05	26.1	0.56	.	.	4.0E-06
3796287	32.1	male	60	p.Ile102Thr	missense_variant	2.2E-05	26.1	0.56	.	.	4.0E-06
4290735	33.9	female	49	p.Ile102Thr	missense_variant	2.2E-05	26.1	0.56	.	.	4.0E-06
5502107	NA	NA	NA	p.Ile102Thr	missense_variant	2.2E-05	26.1	0.56	.	.	4.0E-06
5577146	23.1	female	41	p.Ile102Thr	missense_variant	2.2E-05	26.1	0.56	.	.	4.0E-06

4926416	32.9	female	54	p.Ile102Asn	missense_variant	2.5E-06	32	0.55	.	.	.
1400304	28.7	female	57	p.Thr112AsnfsTer11	frameshift_variant	7.5E-06	.	.	.	.	.
3971018	NA	NA	NA	p.Thr112AsnfsTer11	frameshift_variant	7.5E-06	.	.	.	.	.
5285585	42.6	female	47	p.Thr112AsnfsTer11	frameshift_variant	7.5E-06	.	.	.	.	.
1986115	33.4	male	56	p.Gln115GlufsTer7	frameshift_variant	1.7E-05	.	.	.	.	4.0E-06
3131891	42.1	male	53	p.Gln115GlufsTer7	frameshift_variant	1.7E-05	.	.	.	.	4.0E-06
4202748	23.6	female	46	p.Gln115GlufsTer7	frameshift_variant	1.7E-05	.	.	.	.	4.0E-06
4455938	28.3	male	49	p.Gln115GlufsTer7	frameshift_variant	1.7E-05	.	.	.	.	4.0E-06
4513989	31.8	male	64	p.Gln115GlufsTer7	frameshift_variant	1.7E-05	.	.	.	.	4.0E-06
4599234	32.0	male	41	p.Gln115GlufsTer7	frameshift_variant	1.7E-05	.	.	.	.	4.0E-06
5303129	25.2	female	54	p.Gln115GlufsTer7	frameshift_variant	1.7E-05	.	.	.	.	4.0E-06
3366000	32.4	female	47	p.Ser116PhefsTer6	frameshift_variant	2.5E-06	.	.	8.0E-06	7.0E-06	.
2997197	27.3	male	60	p.Asp122Tyr	missense_variant	2.5E-06	29.4	0.68	.	.	.
1014576	34.0	female	62	p.Asn123Ser	missense_variant	1.2E-04	25.8	0.42	2.4E-05	2.8E-05	2.8E-05
1149240	NA	male	40	p.Asn123Ser	missense_variant	1.2E-04	25.8	0.42	2.4E-05	2.8E-05	2.8E-05
1173813	35.2	female	51	p.Asn123Ser	missense_variant	1.2E-04	25.8	0.42	2.4E-05	2.8E-05	2.8E-05
1204357	24.7	female	68	p.Asn123Ser	missense_variant	1.2E-04	25.8	0.42	2.4E-05	2.8E-05	2.8E-05
1318559	28.3	male	43	p.Asn123Ser	missense_variant	1.2E-04	25.8	0.42	2.4E-05	2.8E-05	2.8E-05
1337726	NA	NA	NA	p.Asn123Ser	missense_variant	1.2E-04	25.8	0.42	2.4E-05	2.8E-05	2.8E-05
1438116	38.6	female	58	p.Asn123Ser	missense_variant	1.2E-04	25.8	0.42	2.4E-05	2.8E-05	2.8E-05
1578614	NA	NA	NA	p.Asn123Ser	missense_variant	1.2E-04	25.8	0.42	2.4E-05	2.8E-05	2.8E-05
1586480	37.7	male	60	p.Asn123Ser	missense_variant	1.2E-04	25.8	0.42	2.4E-05	2.8E-05	2.8E-05
1913306	23.5	male	64	p.Asn123Ser	missense_variant	1.2E-04	25.8	0.42	2.4E-05	2.8E-05	2.8E-05
1972182	31.7	male	68	p.Asn123Ser	missense_variant	1.2E-04	25.8	0.42	2.4E-05	2.8E-05	2.8E-05
2106186	32.1	male	44	p.Asn123Ser	missense_variant	1.2E-04	25.8	0.42	2.4E-05	2.8E-05	2.8E-05
2278231	21.0	male	61	p.Asn123Ser	missense_variant	1.2E-04	25.8	0.42	2.4E-05	2.8E-05	2.8E-05
2354052	40.6	male	67	p.Asn123Ser	missense_variant	1.2E-04	25.8	0.42	2.4E-05	2.8E-05	2.8E-05
2406181	27.8	male	44	p.Asn123Ser	missense_variant	1.2E-04	25.8	0.42	2.4E-05	2.8E-05	2.8E-05

2593179	NA	NA	NA	p.Asn123Ser	missense_variant	1.2E-04	25.8	0.42	2.4E-05	2.8E-05	2.8E-05
2642762	23.1	female	49	p.Asn123Ser	missense_variant	1.2E-04	25.8	0.42	2.4E-05	2.8E-05	2.8E-05
2891734	27.8	male	55	p.Asn123Ser	missense_variant	1.2E-04	25.8	0.42	2.4E-05	2.8E-05	2.8E-05
2929220	27.1	male	62	p.Asn123Ser	missense_variant	1.2E-04	25.8	0.42	2.4E-05	2.8E-05	2.8E-05
2977270	26.7	male	51	p.Asn123Ser	missense_variant	1.2E-04	25.8	0.42	2.4E-05	2.8E-05	2.8E-05
3078365	24.5	female	56	p.Asn123Ser	missense_variant	1.2E-04	25.8	0.42	2.4E-05	2.8E-05	2.8E-05
3123351	29.5	male	67	p.Asn123Ser	missense_variant	1.2E-04	25.8	0.42	2.4E-05	2.8E-05	2.8E-05
3289867	27.7	male	56	p.Asn123Ser	missense_variant	1.2E-04	25.8	0.42	2.4E-05	2.8E-05	2.8E-05
3326704	24.2	male	42	p.Asn123Ser	missense_variant	1.2E-04	25.8	0.42	2.4E-05	2.8E-05	2.8E-05
3554598	24.9	female	61	p.Asn123Ser	missense_variant	1.2E-04	25.8	0.42	2.4E-05	2.8E-05	2.8E-05
3593439	33.2	female	69	p.Asn123Ser	missense_variant	1.2E-04	25.8	0.42	2.4E-05	2.8E-05	2.8E-05
3776941	24.5	female	69	p.Asn123Ser	missense_variant	1.2E-04	25.8	0.42	2.4E-05	2.8E-05	2.8E-05
3841014	28.8	female	66	p.Asn123Ser	missense_variant	1.2E-04	25.8	0.42	2.4E-05	2.8E-05	2.8E-05
3884296	23.4	female	56	p.Asn123Ser	missense_variant	1.2E-04	25.8	0.42	2.4E-05	2.8E-05	2.8E-05
3899889	NA	NA	NA	p.Asn123Ser	missense_variant	1.2E-04	25.8	0.42	2.4E-05	2.8E-05	2.8E-05
3996263	36.8	female	48	p.Asn123Ser	missense_variant	1.2E-04	25.8	0.42	2.4E-05	2.8E-05	2.8E-05
4009102	25.7	male	65	p.Asn123Ser	missense_variant	1.2E-04	25.8	0.42	2.4E-05	2.8E-05	2.8E-05
4192282	25.3	female	57	p.Asn123Ser	missense_variant	1.2E-04	25.8	0.42	2.4E-05	2.8E-05	2.8E-05
4454101	25.4	male	43	p.Asn123Ser	missense_variant	1.2E-04	25.8	0.42	2.4E-05	2.8E-05	2.8E-05
4608125	29.2	male	66	p.Asn123Ser	missense_variant	1.2E-04	25.8	0.42	2.4E-05	2.8E-05	2.8E-05
4648134	32.5	male	51	p.Asn123Ser	missense_variant	1.2E-04	25.8	0.42	2.4E-05	2.8E-05	2.8E-05
4682485	33.2	female	46	p.Asn123Ser	missense_variant	1.2E-04	25.8	0.42	2.4E-05	2.8E-05	2.8E-05
4747106	24.9	female	59	p.Asn123Ser	missense_variant	1.2E-04	25.8	0.42	2.4E-05	2.8E-05	2.8E-05
5012941	31.0	female	61	p.Asn123Ser	missense_variant	1.2E-04	25.8	0.42	2.4E-05	2.8E-05	2.8E-05
5069288	24.3	male	66	p.Asn123Ser	missense_variant	1.2E-04	25.8	0.42	2.4E-05	2.8E-05	2.8E-05
5173413	30.1	female	59	p.Asn123Ser	missense_variant	1.2E-04	25.8	0.42	2.4E-05	2.8E-05	2.8E-05
5322011	19.0	female	55	p.Asn123Ser	missense_variant	1.2E-04	25.8	0.42	2.4E-05	2.8E-05	2.8E-05
5447107	29.5	female	65	p.Asn123Ser	missense_variant	1.2E-04	25.8	0.42	2.4E-05	2.8E-05	2.8E-05

5464157	25.6	male	53	p.Asn123Ser	missense_variant	1.2E-04	25.8	0.42	2.4E-05	2.8E-05	2.8E-05
5542879	27.5	male	65	p.Asn123Ser	missense_variant	1.2E-04	25.8	0.42	2.4E-05	2.8E-05	2.8E-05
5738528	30.0	male	66	p.Asn123Ser	missense_variant	1.2E-04	25.8	0.42	2.4E-05	2.8E-05	2.8E-05
5931648	25.4	female	47	p.Asn123Ser	missense_variant	1.2E-04	25.8	0.42	2.4E-05	2.8E-05	2.8E-05
1806252	34.4	male	59	p.Ile125Asn	missense_variant	2.2E-05	32	0.48	.	.	.
2328999	41.4	male	52	p.Ile125Asn	missense_variant	2.2E-05	32	0.48	.	.	.
2477917	41.2	male	44	p.Ile125Asn	missense_variant	2.2E-05	32	0.48	.	.	.
2520182	30.4	male	62	p.Ile125Asn	missense_variant	2.2E-05	32	0.48	.	.	.
3805024	NA	NA	NA	p.Ile125Asn	missense_variant	2.2E-05	32	0.48	.	.	.
4398805	23.9	male	57	p.Ile125Asn	missense_variant	2.2E-05	32	0.48	.	.	.
4518658	48.6	male	53	p.Ile125Asn	missense_variant	2.2E-05	32	0.48	.	.	.
5395965	20.7	female	68	p.Ile125Asn	missense_variant	2.2E-05	32	0.48	.	.	.
5637159	32.4	male	61	p.Ile125Asn	missense_variant	2.2E-05	32	0.48	.	.	.
5352750	22.0	female	63	p.Cys130Trp	missense_variant	2.5E-06	27.6	0.45	.	.	.
1957427	35.0	female	54	p.Ser136Pro	missense_variant	1.5E-05	25.4	0.75	.	.	.
3423718	NA	NA	NA	p.Ser136Pro	missense_variant	1.5E-05	25.4	0.75	.	.	.
3468428	30.3	male	58	p.Ser136Pro	missense_variant	1.5E-05	25.4	0.75	.	.	.
4967648	25.3	male	64	p.Ser136Pro	missense_variant	1.5E-05	25.4	0.75	.	.	.
5505751	33.6	male	50	p.Ser136Pro	missense_variant	1.5E-05	25.4	0.75	.	.	.
5556039	NA	NA	NA	p.Ser136Pro	missense_variant	1.5E-05	25.4	0.75	.	.	.
2604288	NA	NA	NA	p.Ser136Phe	missense_variant	2.5E-06	28.1	0.62	.	7.0E-06	.
1100507	34.0	female	49	p.Ile137Thr	missense_variant	9.0E-05	26.8	0.53	4.0E-05	2.8E-05	2.4E-05
1317870	26.2	female	55	p.Ile137Thr	missense_variant	9.0E-05	26.8	0.53	4.0E-05	2.8E-05	2.4E-05
1506789	26.6	female	50	p.Ile137Thr	missense_variant	9.0E-05	26.8	0.53	4.0E-05	2.8E-05	2.4E-05
1617392	NA	NA	NA	p.Ile137Thr	missense_variant	9.0E-05	26.8	0.53	4.0E-05	2.8E-05	2.4E-05
1756981	28.8	male	40	p.Ile137Thr	missense_variant	9.0E-05	26.8	0.53	4.0E-05	2.8E-05	2.4E-05
1761184	32.5	female	60	p.Ile137Thr	missense_variant	9.0E-05	26.8	0.53	4.0E-05	2.8E-05	2.4E-05
1938965	29.2	female	58	p.Ile137Thr	missense_variant	9.0E-05	26.8	0.53	4.0E-05	2.8E-05	2.4E-05

1999428	35.9	female	62	p.Ile137Thr	missense_variant	9.0E-05	26.8	0.53	4.0E-05	2.8E-05	2.4E-05
2327116	23.7	female	45	p.Ile137Thr	missense_variant	9.0E-05	26.8	0.53	4.0E-05	2.8E-05	2.4E-05
2996339	23.7	male	41	p.Ile137Thr	missense_variant	9.0E-05	26.8	0.53	4.0E-05	2.8E-05	2.4E-05
3083837	NA	NA	NA	p.Ile137Thr	missense_variant	9.0E-05	26.8	0.53	4.0E-05	2.8E-05	2.4E-05
3259296	21.3	male	56	p.Ile137Thr	missense_variant	9.0E-05	26.8	0.53	4.0E-05	2.8E-05	2.4E-05
3323996	NA	NA	NA	p.Ile137Thr	missense_variant	9.0E-05	26.8	0.53	4.0E-05	2.8E-05	2.4E-05
3393887	21.8	male	64	p.Ile137Thr	missense_variant	9.0E-05	26.8	0.53	4.0E-05	2.8E-05	2.4E-05
3432813	35.5	male	59	p.Ile137Thr	missense_variant	9.0E-05	26.8	0.53	4.0E-05	2.8E-05	2.4E-05
3453153	NA	NA	NA	p.Ile137Thr	missense_variant	9.0E-05	26.8	0.53	4.0E-05	2.8E-05	2.4E-05
3701636	23.1	male	60	p.Ile137Thr	missense_variant	9.0E-05	26.8	0.53	4.0E-05	2.8E-05	2.4E-05
3805267	23.4	female	50	p.Ile137Thr	missense_variant	9.0E-05	26.8	0.53	4.0E-05	2.8E-05	2.4E-05
3925891	33.5	male	60	p.Ile137Thr	missense_variant	9.0E-05	26.8	0.53	4.0E-05	2.8E-05	2.4E-05
4430865	27.7	female	61	p.Ile137Thr	missense_variant	9.0E-05	26.8	0.53	4.0E-05	2.8E-05	2.4E-05
4478104	23.8	male	56	p.Ile137Thr	missense_variant	9.0E-05	26.8	0.53	4.0E-05	2.8E-05	2.4E-05
4676225	28.6	female	45	p.Ile137Thr	missense_variant	9.0E-05	26.8	0.53	4.0E-05	2.8E-05	2.4E-05
4717645	NA	NA	NA	p.Ile137Thr	missense_variant	9.0E-05	26.8	0.53	4.0E-05	2.8E-05	2.4E-05
4754233	34.5	female	69	p.Ile137Thr	missense_variant	9.0E-05	26.8	0.53	4.0E-05	2.8E-05	2.4E-05
4837703	30.0	female	42	p.Ile137Thr	missense_variant	9.0E-05	26.8	0.53	4.0E-05	2.8E-05	2.4E-05
4945627	29.2	female	44	p.Ile137Thr	missense_variant	9.0E-05	26.8	0.53	4.0E-05	2.8E-05	2.4E-05
5028712	27.5	female	63	p.Ile137Thr	missense_variant	9.0E-05	26.8	0.53	4.0E-05	2.8E-05	2.4E-05
5083430	31.2	female	47	p.Ile137Thr	missense_variant	9.0E-05	26.8	0.53	4.0E-05	2.8E-05	2.4E-05
5092839	38.5	male	42	p.Ile137Thr	missense_variant	9.0E-05	26.8	0.53	4.0E-05	2.8E-05	2.4E-05
5133434	31.8	female	67	p.Ile137Thr	missense_variant	9.0E-05	26.8	0.53	4.0E-05	2.8E-05	2.4E-05
5507645	22.9	female	62	p.Ile137Thr	missense_variant	9.0E-05	26.8	0.53	4.0E-05	2.8E-05	2.4E-05
5574044	21.3	female	52	p.Ile137Thr	missense_variant	9.0E-05	26.8	0.53	4.0E-05	2.8E-05	2.4E-05
5600363	24.9	male	63	p.Ile137Thr	missense_variant	9.0E-05	26.8	0.53	4.0E-05	2.8E-05	2.4E-05
5677778	24.5	male	42	p.Ile137Thr	missense_variant	9.0E-05	26.8	0.53	4.0E-05	2.8E-05	2.4E-05
5779471	36.9	female	62	p.Ile137Thr	missense_variant	9.0E-05	26.8	0.53	4.0E-05	2.8E-05	2.4E-05

5808264	28.2	female	59	p.Ile137Thr	missense_variant	9.0E-05	26.8	0.53	4.0E-05	2.8E-05	2.4E-05
1143230	31.1	male	61	p.Cys138Phe	missense_variant	2.5E-06	24.2	0.45	.	.	4.0E-06
4905816	47.4	female	47	p.Val145Ala	missense_variant	2.5E-06	26.3	0.42	.	.	4.0E-06
1190778	28.3	male	57	p.Asp146Ala	missense_variant	2.5E-06	28.6	0.87	.	.	.
3710129	28.8	male	62	p.Asp146Gly	missense_variant	2.5E-06	28.8	0.92	.	.	.
3805957	44.3	female	57	p.Asp146LeufsTer11	frameshift_variant	2.5E-06	.	.	.	.	4.0E-06
1241066	26.1	male	45	p.His158Arg	missense_variant	3.7E-05	25.6	0.61	1.6E-05	3.5E-05	4.0E-06
1380863	25.2	female	52	p.His158Arg	missense_variant	3.7E-05	25.6	0.61	1.6E-05	3.5E-05	4.0E-06
1384021	27.7	male	59	p.His158Arg	missense_variant	3.7E-05	25.6	0.61	1.6E-05	3.5E-05	4.0E-06
1412955	28.5	female	56	p.His158Arg	missense_variant	3.7E-05	25.6	0.61	1.6E-05	3.5E-05	4.0E-06
1570881	25.8	female	62	p.His158Arg	missense_variant	3.7E-05	25.6	0.61	1.6E-05	3.5E-05	4.0E-06
1743275	25.3	male	47	p.His158Arg	missense_variant	3.7E-05	25.6	0.61	1.6E-05	3.5E-05	4.0E-06
2440464	23.7	male	68	p.His158Arg	missense_variant	3.7E-05	25.6	0.61	1.6E-05	3.5E-05	4.0E-06
3103870	30.2	male	43	p.His158Arg	missense_variant	3.7E-05	25.6	0.61	1.6E-05	3.5E-05	4.0E-06
3644525	NA	NA	NA	p.His158Arg	missense_variant	3.7E-05	25.6	0.61	1.6E-05	3.5E-05	4.0E-06
5180932	NA	NA	NA	p.His158Arg	missense_variant	3.7E-05	25.6	0.61	1.6E-05	3.5E-05	4.0E-06
5287608	36.0	male	62	p.His158Arg	missense_variant	3.7E-05	25.6	0.61	1.6E-05	3.5E-05	4.0E-06
5307414	24.9	female	53	p.His158Arg	missense_variant	3.7E-05	25.6	0.61	1.6E-05	3.5E-05	4.0E-06
5357498	23.5	male	45	p.His158Arg	missense_variant	3.7E-05	25.6	0.61	1.6E-05	3.5E-05	4.0E-06
5556183	19.3	female	63	p.His158Arg	missense_variant	3.7E-05	25.6	0.61	1.6E-05	3.5E-05	4.0E-06
5736298	20.0	female	46	p.His158Arg	missense_variant	3.7E-05	25.6	0.61	1.6E-05	3.5E-05	4.0E-06
3968759	NA	NA	NA	p.Ile160Val	missense_variant	2.5E-06	27.4	0.37	.	.	.
1249021	22.4	female	57	p.Met161Thr	missense_variant	5.0E-06	28.4	0.49	.	.	4.0E-06
4523416	NA	NA	NA	p.Met161Thr	missense_variant	5.0E-06	28.4	0.49	.	.	4.0E-06
1054215	30.9	female	65	p.Arg165Trp	missense_variant	5.0E-05	31	0.50	.	1.4E-05	2.0E-05
1176128	27.9	male	47	p.Arg165Trp	missense_variant	5.0E-05	31	0.50	.	1.4E-05	2.0E-05
1193660	37.8	female	60	p.Arg165Trp	missense_variant	5.0E-05	31	0.50	.	1.4E-05	2.0E-05
1622359	NA	NA	NA	p.Arg165Trp	missense_variant	5.0E-05	31	0.50	.	1.4E-05	2.0E-05

1728366	23.9	female	45	p.Arg165Trp	missense_variant	5.0E-05	31	0.50	.	1.4E-05	2.0E-05
2405729	30.7	male	61	p.Arg165Trp	missense_variant	5.0E-05	31	0.50	.	1.4E-05	2.0E-05
2500178	30.3	male	53	p.Arg165Trp	missense_variant	5.0E-05	31	0.50	.	1.4E-05	2.0E-05
2600330	38.2	female	50	p.Arg165Trp	missense_variant	5.0E-05	31	0.50	.	1.4E-05	2.0E-05
2676034	33.7	male	54	p.Arg165Trp	missense_variant	5.0E-05	31	0.50	.	1.4E-05	2.0E-05
3095787	26.8	male	64	p.Arg165Trp	missense_variant	5.0E-05	31	0.50	.	1.4E-05	2.0E-05
3163934	NA	NA	NA	p.Arg165Trp	missense_variant	5.0E-05	31	0.50	.	1.4E-05	2.0E-05
3195944	24.2	female	56	p.Arg165Trp	missense_variant	5.0E-05	31	0.50	.	1.4E-05	2.0E-05
3764277	22.0	male	46	p.Arg165Trp	missense_variant	5.0E-05	31	0.50	.	1.4E-05	2.0E-05
3768536	29.0	male	53	p.Arg165Trp	missense_variant	5.0E-05	31	0.50	.	1.4E-05	2.0E-05
4907573	30.3	male	70	p.Arg165Trp	missense_variant	5.0E-05	31	0.50	.	1.4E-05	2.0E-05
5088879	30.8	female	43	p.Arg165Trp	missense_variant	5.0E-05	31	0.50	.	1.4E-05	2.0E-05
5148818	36.1	female	57	p.Arg165Trp	missense_variant	5.0E-05	31	0.50	.	1.4E-05	2.0E-05
5225941	33.6	female	65	p.Arg165Trp	missense_variant	5.0E-05	31	0.50	.	1.4E-05	2.0E-05
5267594	37.4	female	57	p.Arg165Trp	missense_variant	5.0E-05	31	0.50	.	1.4E-05	2.0E-05
5900935	25.6	female	61	p.Arg165Trp	missense_variant	5.0E-05	31	0.50	.	1.4E-05	2.0E-05
1239203	27.9	female	55	p.Arg165Gln	missense_variant	4.2E-05	31	0.67	1.6E-05	1.4E-05	2.4E-05
1385148	NA	NA	NA	p.Arg165Gln	missense_variant	4.2E-05	31	0.67	1.6E-05	1.4E-05	2.4E-05
1406409	27.7	male	40	p.Arg165Gln	missense_variant	4.2E-05	31	0.67	1.6E-05	1.4E-05	2.4E-05
1773490	NA	NA	NA	p.Arg165Gln	missense_variant	4.2E-05	31	0.67	1.6E-05	1.4E-05	2.4E-05
1800525	25.2	female	63	p.Arg165Gln	missense_variant	4.2E-05	31	0.67	1.6E-05	1.4E-05	2.4E-05
2472518	26.4	female	69	p.Arg165Gln	missense_variant	4.2E-05	31	0.67	1.6E-05	1.4E-05	2.4E-05
2573090	29.0	male	55	p.Arg165Gln	missense_variant	4.2E-05	31	0.67	1.6E-05	1.4E-05	2.4E-05
3225956	NA	NA	NA	p.Arg165Gln	missense_variant	4.2E-05	31	0.67	1.6E-05	1.4E-05	2.4E-05
3567955	37.1	male	66	p.Arg165Gln	missense_variant	4.2E-05	31	0.67	1.6E-05	1.4E-05	2.4E-05
3980563	24.1	male	49	p.Arg165Gln	missense_variant	4.2E-05	31	0.67	1.6E-05	1.4E-05	2.4E-05
4061953	29.0	female	60	p.Arg165Gln	missense_variant	4.2E-05	31	0.67	1.6E-05	1.4E-05	2.4E-05
4743200	36.9	female	57	p.Arg165Gln	missense_variant	4.2E-05	31	0.67	1.6E-05	1.4E-05	2.4E-05

4867071	35.9	female	56	p.Arg165Gln	missense_variant	4.2E-05	31	0.67	1.6E-05	1.4E-05	2.4E-05
5030780	30.8	male	62	p.Arg165Gln	missense_variant	4.2E-05	31	0.67	1.6E-05	1.4E-05	2.4E-05
5117609	30.5	male	62	p.Arg165Gln	missense_variant	4.2E-05	31	0.67	1.6E-05	1.4E-05	2.4E-05
5263549	NA	NA	NA	p.Arg165Gln	missense_variant	4.2E-05	31	0.67	1.6E-05	1.4E-05	2.4E-05
6018515	19.4	female	57	p.Arg165Gln	missense_variant	4.2E-05	31	0.67	1.6E-05	1.4E-05	2.4E-05
5704494	24.8	male	55	p.Ile173Thr	missense_variant	2.5E-06	29.4	0.82	.	.	.
5903169	29.4	male	63	p.Trp174Arg	missense_variant	2.5E-06	33	0.92	.	.	.
2889776	25.7	female	42	p.Ser180Pro	missense_variant	1.0E-05	21.9	0.59	8.0E-06	.	4.0E-06
3757603	39.8	female	43	p.Ser180Pro	missense_variant	1.0E-05	21.9	0.59	8.0E-06	.	4.0E-06
3838438	23.9	male	49	p.Ser180Pro	missense_variant	1.0E-05	21.9	0.59	8.0E-06	.	4.0E-06
5239039	25.4	male	62	p.Ser180Pro	missense_variant	1.0E-05	21.9	0.59	8.0E-06	.	4.0E-06
3629050	26.5	female	67	p.Gly181Asp	missense_variant	2.5E-06	23.2	0.67	.	.	1.6E-05
2142760	27.3	male	67	p.Leu183Phe	missense_variant	7.5E-06	22.3	0.35	.	.	.
3622748	26.9	male	66	p.Leu183Phe	missense_variant	7.5E-06	22.3	0.35	.	.	.
3964567	28.6	male	66	p.Leu183Phe	missense_variant	7.5E-06	22.3	0.35	.	.	.
3155058	23.6	male	62	p.Phe184Ile	missense_variant	5.0E-06	25.6	0.62	.	.	.
4753327	26.4	male	59	p.Phe184Ile	missense_variant	5.0E-06	25.6	0.62	.	.	.
1733621	32.5	female	65	p.Tyr187ThrfsTer31	frameshift_variant	2.5E-06	.	.	.	.	4.0E-06
4894925	28.6	male	62	p.Cys196Tyr	missense_variant	2.5E-06	26.1	0.95	.	.	4.0E-06
2082701	24.9	female	68	p.Met215Ile	missense_variant	1.3E-05	25.2	0.43	.	7.0E-06	8.0E-06
2241509	27.6	male	48	p.Met215Ile	missense_variant	1.3E-05	25.2	0.43	.	7.0E-06	8.0E-06
4064417	31.9	female	56	p.Met215Ile	missense_variant	1.3E-05	25.2	0.43	.	7.0E-06	8.0E-06
4885862	32.0	female	61	p.Met215Ile	missense_variant	1.3E-05	25.2	0.43	.	7.0E-06	8.0E-06
5412607	26.3	male	43	p.Met215Ile	missense_variant	1.3E-05	25.2	0.43	.	7.0E-06	8.0E-06
1878615	21.1	male	61	p.Met215Val	missense_variant	5.0E-06	27.1	0.42	.	.	.
1934678	40.7	male	66	p.Met215Val	missense_variant	5.0E-06	27.1	0.42	.	.	.
4857870	20.3	female	59	p.Met218Thr	missense_variant	2.5E-06	25.3	0.38	1.6E-05	7.0E-06	4.0E-05
3774034	26.7	male	68	p.Ala219Ser	missense_variant	2.5E-06	28.2	0.55	.	.	.

1642332	32.9	female	65	p.Met241Thr	missense_variant	5.0E-06	27.8	0.65	.	.	.
3558939	30.1	male	69	p.Met241Thr	missense_variant	5.0E-06	27.8	0.65	.	.	.
1267284	26.3	male	52	p.Ala244Val	missense_variant	4.0E-05	27.1	0.68	2.4E-05	2.8E-05	2.8E-05
1891091	33.5	female	59	p.Ala244Val	missense_variant	4.0E-05	27.1	0.68	2.4E-05	2.8E-05	2.8E-05
2334031	21.8	female	62	p.Ala244Val	missense_variant	4.0E-05	27.1	0.68	2.4E-05	2.8E-05	2.8E-05
2550476	25.6	female	65	p.Ala244Val	missense_variant	4.0E-05	27.1	0.68	2.4E-05	2.8E-05	2.8E-05
2681077	21.3	female	53	p.Ala244Val	missense_variant	4.0E-05	27.1	0.68	2.4E-05	2.8E-05	2.8E-05
2763902	31.7	female	57	p.Ala244Val	missense_variant	4.0E-05	27.1	0.68	2.4E-05	2.8E-05	2.8E-05
3124616	31.4	female	63	p.Ala244Val	missense_variant	4.0E-05	27.1	0.68	2.4E-05	2.8E-05	2.8E-05
3673556	23.6	male	66	p.Ala244Val	missense_variant	4.0E-05	27.1	0.68	2.4E-05	2.8E-05	2.8E-05
3726681	25.4	male	67	p.Ala244Val	missense_variant	4.0E-05	27.1	0.68	2.4E-05	2.8E-05	2.8E-05
4054380	NA	NA	NA	p.Ala244Val	missense_variant	4.0E-05	27.1	0.68	2.4E-05	2.8E-05	2.8E-05
4563640	27.3	female	54	p.Ala244Val	missense_variant	4.0E-05	27.1	0.68	2.4E-05	2.8E-05	2.8E-05
4672856	23.3	female	53	p.Ala244Val	missense_variant	4.0E-05	27.1	0.68	2.4E-05	2.8E-05	2.8E-05
4953626	37.0	male	58	p.Ala244Val	missense_variant	4.0E-05	27.1	0.68	2.4E-05	2.8E-05	2.8E-05
5218978	27.1	male	40	p.Ala244Val	missense_variant	4.0E-05	27.1	0.68	2.4E-05	2.8E-05	2.8E-05
5386607	24.2	female	62	p.Ala244Val	missense_variant	4.0E-05	27.1	0.68	2.4E-05	2.8E-05	2.8E-05
5496670	21.6	female	58	p.Ala244Val	missense_variant	4.0E-05	27.1	0.68	2.4E-05	2.8E-05	2.8E-05
4041245	33.6	female	62	p.Ala244Glu	missense_variant	5.0E-06	27	0.81	2.4E-05	7.0E-06	8.0E-06
5400068	NA	NA	NA	p.Ala244Glu	missense_variant	5.0E-06	27	0.81	2.4E-05	7.0E-06	8.0E-06
1214667	33.5	male	69	p.Leu250Gln	missense_variant	1.7E-05	29.9	0.76	.	7.0E-06	1.6E-05
2066279	27.6	female	61	p.Leu250Gln	missense_variant	1.7E-05	29.9	0.76	.	7.0E-06	1.6E-05
2352734	23.6	female	52	p.Leu250Gln	missense_variant	1.7E-05	29.9	0.76	.	7.0E-06	1.6E-05
3555130	23.8	female	52	p.Leu250Gln	missense_variant	1.7E-05	29.9	0.76	.	7.0E-06	1.6E-05
3736171	23.3	male	63	p.Leu250Gln	missense_variant	1.7E-05	29.9	0.76	.	7.0E-06	1.6E-05
5110595	26.0	male	64	p.Leu250Gln	missense_variant	1.7E-05	29.9	0.76	.	7.0E-06	1.6E-05
5424419	36.7	male	61	p.Leu250Gln	missense_variant	1.7E-05	29.9	0.76	.	7.0E-06	1.6E-05
1046614	25.6	male	62	p.Ile251TrpfsTer34	frameshift_variant	3.5E-05	.	.	.	2.8E-05	3.6E-05

1211620	37.1	male	61	p.Ile251TrpfsTer34	frameshift_variant	3.5E-05	.	.	.	2.8E-05	3.6E-05
1312906	30.8	male	58	p.Ile251TrpfsTer34	frameshift_variant	3.5E-05	.	.	.	2.8E-05	3.6E-05
1549731	NA	NA	NA	p.Ile251TrpfsTer34	frameshift_variant	3.5E-05	.	.	.	2.8E-05	3.6E-05
1933006	32.0	male	61	p.Ile251TrpfsTer34	frameshift_variant	3.5E-05	.	.	.	2.8E-05	3.6E-05
2351823	31.7	female	62	p.Ile251TrpfsTer34	frameshift_variant	3.5E-05	.	.	.	2.8E-05	3.6E-05
2393054	33.7	male	62	p.Ile251TrpfsTer34	frameshift_variant	3.5E-05	.	.	.	2.8E-05	3.6E-05
3155252	24.3	female	48	p.Ile251TrpfsTer34	frameshift_variant	3.5E-05	.	.	.	2.8E-05	3.6E-05
3255516	27.3	female	40	p.Ile251TrpfsTer34	frameshift_variant	3.5E-05	.	.	.	2.8E-05	3.6E-05
3417824	24.4	male	53	p.Ile251TrpfsTer34	frameshift_variant	3.5E-05	.	.	.	2.8E-05	3.6E-05
3684198	32.5	male	52	p.Ile251TrpfsTer34	frameshift_variant	3.5E-05	.	.	.	2.8E-05	3.6E-05
4356368	26.8	male	65	p.Ile251TrpfsTer34	frameshift_variant	3.5E-05	.	.	.	2.8E-05	3.6E-05
4761722	26.5	male	56	p.Ile251TrpfsTer34	frameshift_variant	3.5E-05	.	.	.	2.8E-05	3.6E-05
5499707	NA	NA	NA	p.Ile251TrpfsTer34	frameshift_variant	3.5E-05	.	.	.	2.8E-05	3.6E-05
1014534	30.9	female	67	p.Gly252Ser	missense_variant	1.4E-04	25.9	0.82	5.6E-05	7.7E-05	4.0E-05
1068328	28.8	female	67	p.Gly252Ser	missense_variant	1.4E-04	25.9	0.82	5.6E-05	7.7E-05	4.0E-05
1078482	31.6	male	57	p.Gly252Ser	missense_variant	1.4E-04	25.9	0.82	5.6E-05	7.7E-05	4.0E-05
1187634	25.1	female	61	p.Gly252Ser	missense_variant	1.4E-04	25.9	0.82	5.6E-05	7.7E-05	4.0E-05
1245951	25.4	male	44	p.Gly252Ser	missense_variant	1.4E-04	25.9	0.82	5.6E-05	7.7E-05	4.0E-05
1318994	31.9	male	60	p.Gly252Ser	missense_variant	1.4E-04	25.9	0.82	5.6E-05	7.7E-05	4.0E-05
1495579	27.4	male	60	p.Gly252Ser	missense_variant	1.4E-04	25.9	0.82	5.6E-05	7.7E-05	4.0E-05
1601568	27.0	male	61	p.Gly252Ser	missense_variant	1.4E-04	25.9	0.82	5.6E-05	7.7E-05	4.0E-05
1698228	26.3	male	66	p.Gly252Ser	missense_variant	1.4E-04	25.9	0.82	5.6E-05	7.7E-05	4.0E-05
1739448	28.1	male	67	p.Gly252Ser	missense_variant	1.4E-04	25.9	0.82	5.6E-05	7.7E-05	4.0E-05
1789865	32.0	female	63	p.Gly252Ser	missense_variant	1.4E-04	25.9	0.82	5.6E-05	7.7E-05	4.0E-05
1793829	27.1	female	58	p.Gly252Ser	missense_variant	1.4E-04	25.9	0.82	5.6E-05	7.7E-05	4.0E-05
1861300	24.7	male	44	p.Gly252Ser	missense_variant	1.4E-04	25.9	0.82	5.6E-05	7.7E-05	4.0E-05
1947013	25.7	male	66	p.Gly252Ser	missense_variant	1.4E-04	25.9	0.82	5.6E-05	7.7E-05	4.0E-05
1983251	26.1	male	59	p.Gly252Ser	missense_variant	1.4E-04	25.9	0.82	5.6E-05	7.7E-05	4.0E-05

2251960	23.0	female	54	p.Gly252Ser	missense_variant	1.4E-04	25.9	0.82	5.6E-05	7.7E-05	4.0E-05
2256366	24.5	NA	65	p.Gly252Ser	missense_variant	1.4E-04	25.9	0.82	5.6E-05	7.7E-05	4.0E-05
2360113	25.7	male	49	p.Gly252Ser	missense_variant	1.4E-04	25.9	0.82	5.6E-05	7.7E-05	4.0E-05
2397908	24.1	female	57	p.Gly252Ser	missense_variant	1.4E-04	25.9	0.82	5.6E-05	7.7E-05	4.0E-05
2572730	22.7	female	68	p.Gly252Ser	missense_variant	1.4E-04	25.9	0.82	5.6E-05	7.7E-05	4.0E-05
2744870	27.7	male	40	p.Gly252Ser	missense_variant	1.4E-04	25.9	0.82	5.6E-05	7.7E-05	4.0E-05
2782577	25.1	female	44	p.Gly252Ser	missense_variant	1.4E-04	25.9	0.82	5.6E-05	7.7E-05	4.0E-05
2794986	24.7	male	70	p.Gly252Ser	missense_variant	1.4E-04	25.9	0.82	5.6E-05	7.7E-05	4.0E-05
2837654	27.6	female	45	p.Gly252Ser	missense_variant	1.4E-04	25.9	0.82	5.6E-05	7.7E-05	4.0E-05
3056478	28.0	female	68	p.Gly252Ser	missense_variant	1.4E-04	25.9	0.82	5.6E-05	7.7E-05	4.0E-05
3067457	26.1	male	70	p.Gly252Ser	missense_variant	1.4E-04	25.9	0.82	5.6E-05	7.7E-05	4.0E-05
3100688	31.8	female	53	p.Gly252Ser	missense_variant	1.4E-04	25.9	0.82	5.6E-05	7.7E-05	4.0E-05
3182501	NA	NA	NA	p.Gly252Ser	missense_variant	1.4E-04	25.9	0.82	5.6E-05	7.7E-05	4.0E-05
3201757	29.0	male	60	p.Gly252Ser	missense_variant	1.4E-04	25.9	0.82	5.6E-05	7.7E-05	4.0E-05
3206184	29.7	male	50	p.Gly252Ser	missense_variant	1.4E-04	25.9	0.82	5.6E-05	7.7E-05	4.0E-05
3893594	28.6	male	70	p.Gly252Ser	missense_variant	1.4E-04	25.9	0.82	5.6E-05	7.7E-05	4.0E-05
4081183	30.1	male	57	p.Gly252Ser	missense_variant	1.4E-04	25.9	0.82	5.6E-05	7.7E-05	4.0E-05
4084977	23.0	female	66	p.Gly252Ser	missense_variant	1.4E-04	25.9	0.82	5.6E-05	7.7E-05	4.0E-05
4166149	23.2	female	69	p.Gly252Ser	missense_variant	1.4E-04	25.9	0.82	5.6E-05	7.7E-05	4.0E-05
4248852	24.3	female	64	p.Gly252Ser	missense_variant	1.4E-04	25.9	0.82	5.6E-05	7.7E-05	4.0E-05
4257062	25.6	female	66	p.Gly252Ser	missense_variant	1.4E-04	25.9	0.82	5.6E-05	7.7E-05	4.0E-05
4417370	21.1	male	64	p.Gly252Ser	missense_variant	1.4E-04	25.9	0.82	5.6E-05	7.7E-05	4.0E-05
4434801	27.9	female	63	p.Gly252Ser	missense_variant	1.4E-04	25.9	0.82	5.6E-05	7.7E-05	4.0E-05
4437155	30.0	female	46	p.Gly252Ser	missense_variant	1.4E-04	25.9	0.82	5.6E-05	7.7E-05	4.0E-05
4447228	31.8	male	41	p.Gly252Ser	missense_variant	1.4E-04	25.9	0.82	5.6E-05	7.7E-05	4.0E-05
4513677	30.4	male	62	p.Gly252Ser	missense_variant	1.4E-04	25.9	0.82	5.6E-05	7.7E-05	4.0E-05
4541212	28.3	female	60	p.Gly252Ser	missense_variant	1.4E-04	25.9	0.82	5.6E-05	7.7E-05	4.0E-05
4548603	32.0	male	61	p.Gly252Ser	missense_variant	1.4E-04	25.9	0.82	5.6E-05	7.7E-05	4.0E-05

4783476	23.2	male	67	p.Gly252Ser	missense_variant	1.4E-04	25.9	0.82	5.6E-05	7.7E-05	4.0E-05
4989575	25.8	female	50	p.Gly252Ser	missense_variant	1.4E-04	25.9	0.82	5.6E-05	7.7E-05	4.0E-05
5161598	25.5	female	59	p.Gly252Ser	missense_variant	1.4E-04	25.9	0.82	5.6E-05	7.7E-05	4.0E-05
5403740	31.2	female	63	p.Gly252Ser	missense_variant	1.4E-04	25.9	0.82	5.6E-05	7.7E-05	4.0E-05
5446395	23.7	female	42	p.Gly252Ser	missense_variant	1.4E-04	25.9	0.82	5.6E-05	7.7E-05	4.0E-05
5543588	25.4	male	68	p.Gly252Ser	missense_variant	1.4E-04	25.9	0.82	5.6E-05	7.7E-05	4.0E-05
5619796	26.9	male	64	p.Gly252Ser	missense_variant	1.4E-04	25.9	0.82	5.6E-05	7.7E-05	4.0E-05
5678136	27.3	male	64	p.Gly252Ser	missense_variant	1.4E-04	25.9	0.82	5.6E-05	7.7E-05	4.0E-05
5724023	25.9	male	46	p.Gly252Ser	missense_variant	1.4E-04	25.9	0.82	5.6E-05	7.7E-05	4.0E-05
5797153	23.9	female	46	p.Gly252Ser	missense_variant	1.4E-04	25.9	0.82	5.6E-05	7.7E-05	4.0E-05
5877990	30.7	female	59	p.Gly252Ser	missense_variant	1.4E-04	25.9	0.82	5.6E-05	7.7E-05	4.0E-05
5975002	20.8	female	42	p.Gly252Ser	missense_variant	1.4E-04	25.9	0.82	5.6E-05	7.7E-05	4.0E-05
1226066	NA	NA	NA	p.Trp258Ter	stop_gained	7.5E-06	41	.	.	.	.
1353264	33.8	male	62	p.Trp258Ter	stop_gained	7.5E-06	41	.	.	.	.
2107110	42.2	male	62	p.Trp258Ter	stop_gained	7.5E-06	41	.	.	.	.
1318429	27.4	male	59	p.Cys271Tyr	missense_variant	3.0E-05	25.7	0.84	.	.	.
1573883	19.1	male	55	p.Cys271Tyr	missense_variant	3.0E-05	25.7	0.84	.	.	.
1651427	28.5	female	66	p.Cys271Tyr	missense_variant	3.0E-05	25.7	0.84	.	.	.
2584334	32.6	male	66	p.Cys271Tyr	missense_variant	3.0E-05	25.7	0.84	.	.	.
2932881	28.0	male	46	p.Cys271Tyr	missense_variant	3.0E-05	25.7	0.84	.	.	.
3311591	NA	NA	NA	p.Cys271Tyr	missense_variant	3.0E-05	25.7	0.84	.	.	.
3854947	40.3	female	63	p.Cys271Tyr	missense_variant	3.0E-05	25.7	0.84	.	.	.
4311208	28.6	male	65	p.Cys271Tyr	missense_variant	3.0E-05	25.7	0.84	.	.	.
4660457	24.5	male	44	p.Cys271Tyr	missense_variant	3.0E-05	25.7	0.84	.	.	.
5123898	35.6	female	59	p.Cys271Tyr	missense_variant	3.0E-05	25.7	0.84	.	.	.
5160780	25.9	female	67	p.Cys271Tyr	missense_variant	3.0E-05	25.7	0.84	.	.	.
5597794	40.3	male	47	p.Cys271Tyr	missense_variant	3.0E-05	25.7	0.84	.	.	.
1485299	NA	NA	NA	p.Cys271Arg	missense_variant	2.5E-06	29.7	0.84	.	.	4.0E-06

2248353	23.0	male	51	p.Asn274Ser	missense_variant	2.5E-06	24.3	0.56	8.0E-06	7.0E-06	8.0E-06
3004434	28.0	male	43	p.Pro275Ser	missense_variant	5.0E-06	25.4	0.69	.	7.0E-06	2.4E-05
4538110	29.6	female	42	p.Pro275Ser	missense_variant	5.0E-06	25.4	0.69	.	7.0E-06	2.4E-05
1547103	33.1	male	49	p.Tyr276Ter	stop_gained	5.0E-06	.	.	8.0E-06	7.0E-06	.
5411740	26.9	male	42	p.Tyr276Ter	stop_gained	5.0E-06	.	.	8.0E-06	7.0E-06	.
1129052	35.5	male	45	p.Phe280AlafsTer12	frameshift_variant	2.5E-05	.	.	1.6E-05	.	4.0E-06
1374843	26.3	male	65	p.Phe280AlafsTer12	frameshift_variant	2.5E-05	.	.	1.6E-05	.	4.0E-06
1422118	29.2	male	60	p.Phe280AlafsTer12	frameshift_variant	2.5E-05	.	.	1.6E-05	.	4.0E-06
1848479	28.6	female	45	p.Phe280AlafsTer12	frameshift_variant	2.5E-05	.	.	1.6E-05	.	4.0E-06
3423651	NA	NA	NA	p.Phe280AlafsTer12	frameshift_variant	2.5E-05	.	.	1.6E-05	.	4.0E-06
3590658	25.2	male	64	p.Phe280AlafsTer12	frameshift_variant	2.5E-05	.	.	1.6E-05	.	4.0E-06
3788842	30.7	female	66	p.Phe280AlafsTer12	frameshift_variant	2.5E-05	.	.	1.6E-05	.	4.0E-06
4747364	28.9	female	62	p.Phe280AlafsTer12	frameshift_variant	2.5E-05	.	.	1.6E-05	.	4.0E-06
4877811	27.3	female	54	p.Phe280AlafsTer12	frameshift_variant	2.5E-05	.	.	1.6E-05	.	4.0E-06
5298846	26.4	male	51	p.Phe280AlafsTer12	frameshift_variant	2.5E-05	.	.	1.6E-05	.	4.0E-06
4336507	23.8	female	50	p.Phe284Cys	missense_variant	7.5E-06	32	0.77	.	.	4.0E-06
4761238	28.5	male	42	p.Phe284Cys	missense_variant	7.5E-06	32	0.77	.	.	4.0E-06
5490331	23.1	male	65	p.Phe284Cys	missense_variant	7.5E-06	32	0.77	.	.	4.0E-06
2805541	39.1	female	57	p.Phe284Ile	missense_variant	2.5E-06	32	0.76	.	.	.
3129619	NA	NA	NA	p.Met292SerfsTer9	frameshift_variant	2.5E-06	.	.	.	.	8.0E-06
1117178	28.0	male	63	p.Ser295Pro	missense_variant	1.5E-05	28	0.84	2.4E-05	1.4E-05	1.2E-05
1280931	30.4	female	59	p.Ser295Pro	missense_variant	1.5E-05	28	0.84	2.4E-05	1.4E-05	1.2E-05
3628269	26.3	female	49	p.Ser295Pro	missense_variant	1.5E-05	28	0.84	2.4E-05	1.4E-05	1.2E-05
5412957	22.9	female	51	p.Ser295Pro	missense_variant	1.5E-05	28	0.84	2.4E-05	1.4E-05	1.2E-05
5846023	25.2	male	48	p.Ser295Pro	missense_variant	1.5E-05	28	0.84	2.4E-05	1.4E-05	1.2E-05
5995066	19.9	female	68	p.Ser295Pro	missense_variant	1.5E-05	28	0.84	2.4E-05	1.4E-05	1.2E-05
1170669	31.5	male	59	p.Ser295Ter	stop_gained	1.5E-05	47	.	.	.	.
4418202	31.7	male	51	p.Ser295Ter	stop_gained	1.5E-05	47	.	.	.	.

4776228	28.5	male	50	p.Ser295Ter	stop_gained	1.5E-05	47	.	.	.	.
4782939	27.6	male	57	p.Ser295Ter	stop_gained	1.5E-05	47	.	.	.	.
5260783	32.1	female	63	p.Ser295Ter	stop_gained	1.5E-05	47	.	.	.	.
5781140	27.3	male	63	p.Ser295Ter	stop_gained	1.5E-05	47	.	.	.	.
5165152	23.7	male	47	p.Ser295Leu	missense_variant	2.5E-06	34	0.71	.	.	.
2766605	42.5	female	56	p.Pro299His	missense_variant	1.5E-05	29.7	0.95	4.0E-05	4.2E-05	8.0E-06
3908180	33.6	male	66	p.Pro299His	missense_variant	1.5E-05	29.7	0.95	4.0E-05	4.2E-05	8.0E-06
4114299	33.2	female	64	p.Pro299His	missense_variant	1.5E-05	29.7	0.95	4.0E-05	4.2E-05	8.0E-06
4457674	32.4	male	63	p.Pro299His	missense_variant	1.5E-05	29.7	0.95	4.0E-05	4.2E-05	8.0E-06
4632201	24.5	female	57	p.Pro299His	missense_variant	1.5E-05	29.7	0.95	4.0E-05	4.2E-05	8.0E-06
5282683	30.7	female	65	p.Pro299His	missense_variant	1.5E-05	29.7	0.95	4.0E-05	4.2E-05	8.0E-06
2097896	NA	NA	NA	p.Tyr302Phe	missense_variant	5.0E-06	27.4	0.90	.	.	8.0E-06
5204419	26.4	male	65	p.Tyr302Phe	missense_variant	5.0E-06	27.4	0.90	.	.	8.0E-06
1824409	26.7	male	63	p.Arg305Gln	missense_variant	1.7E-05	32	0.54	3.2E-05	2.8E-05	3.6E-05
2940832	27.1	male	55	p.Arg305Gln	missense_variant	1.7E-05	32	0.54	3.2E-05	2.8E-05	3.6E-05
4273905	34.5	male	46	p.Arg305Gln	missense_variant	1.7E-05	32	0.54	3.2E-05	2.8E-05	3.6E-05
4688918	20.8	male	56	p.Arg305Gln	missense_variant	1.7E-05	32	0.54	3.2E-05	2.8E-05	3.6E-05
4807525	49.0	female	60	p.Arg305Gln	missense_variant	1.7E-05	32	0.54	3.2E-05	2.8E-05	3.6E-05
4924291	26.0	male	61	p.Arg305Gln	missense_variant	1.7E-05	32	0.54	3.2E-05	2.8E-05	3.6E-05
5085759	NA	NA	NA	p.Arg305Gln	missense_variant	1.7E-05	32	0.54	3.2E-05	2.8E-05	3.6E-05
1207444	39.9	male	68	p.Arg305Trp	missense_variant	1.0E-05	27	0.39	4.0E-05	4.9E-05	2.8E-05
3183654	28.0	male	41	p.Arg305Trp	missense_variant	1.0E-05	27	0.39	4.0E-05	4.9E-05	2.8E-05
4753652	31.1	female	45	p.Arg305Trp	missense_variant	1.0E-05	27	0.39	4.0E-05	4.9E-05	2.8E-05
5158480	21.6	female	59	p.Arg305Trp	missense_variant	1.0E-05	27	0.39	4.0E-05	4.9E-05	2.8E-05
1159403	26.8	male	44	p.Ser306Asn	missense_variant	5.0E-05	25.4	0.43	3.2E-05	2.1E-05	8.0E-06
1215998	39.1	female	61	p.Ser306Asn	missense_variant	5.0E-05	25.4	0.43	3.2E-05	2.1E-05	8.0E-06
1228107	26.3	male	67	p.Ser306Asn	missense_variant	5.0E-05	25.4	0.43	3.2E-05	2.1E-05	8.0E-06
1396128	19.6	female	51	p.Ser306Asn	missense_variant	5.0E-05	25.4	0.43	3.2E-05	2.1E-05	8.0E-06

1448695	NA	female	69	p.Ser306Asn	missense_variant	5.0E-05	25.4	0.43	3.2E-05	2.1E-05	8.0E-06
1617038	42.7	female	54	p.Ser306Asn	missense_variant	5.0E-05	25.4	0.43	3.2E-05	2.1E-05	8.0E-06
1810409	NA	NA	NA	p.Ser306Asn	missense_variant	5.0E-05	25.4	0.43	3.2E-05	2.1E-05	8.0E-06
2097483	55.1	female	50	p.Ser306Asn	missense_variant	5.0E-05	25.4	0.43	3.2E-05	2.1E-05	8.0E-06
2221009	NA	NA	NA	p.Ser306Asn	missense_variant	5.0E-05	25.4	0.43	3.2E-05	2.1E-05	8.0E-06
3261670	27.8	male	59	p.Ser306Asn	missense_variant	5.0E-05	25.4	0.43	3.2E-05	2.1E-05	8.0E-06
3642712	33.2	female	67	p.Ser306Asn	missense_variant	5.0E-05	25.4	0.43	3.2E-05	2.1E-05	8.0E-06
3700568	30.4	male	53	p.Ser306Asn	missense_variant	5.0E-05	25.4	0.43	3.2E-05	2.1E-05	8.0E-06
3799480	21.0	male	62	p.Ser306Asn	missense_variant	5.0E-05	25.4	0.43	3.2E-05	2.1E-05	8.0E-06
4346533	NA	NA	NA	p.Ser306Asn	missense_variant	5.0E-05	25.4	0.43	3.2E-05	2.1E-05	8.0E-06
4381306	23.5	male	52	p.Ser306Asn	missense_variant	5.0E-05	25.4	0.43	3.2E-05	2.1E-05	8.0E-06
4391842	33.8	female	51	p.Ser306Asn	missense_variant	5.0E-05	25.4	0.43	3.2E-05	2.1E-05	8.0E-06
4539042	23.3	female	65	p.Ser306Asn	missense_variant	5.0E-05	25.4	0.43	3.2E-05	2.1E-05	8.0E-06
4647624	37.1	male	51	p.Ser306Asn	missense_variant	5.0E-05	25.4	0.43	3.2E-05	2.1E-05	8.0E-06
5352640	19.2	female	56	p.Ser306Asn	missense_variant	5.0E-05	25.4	0.43	3.2E-05	2.1E-05	8.0E-06
5854292	29.5	female	60	p.Ser306Asn	missense_variant	5.0E-05	25.4	0.43	3.2E-05	2.1E-05	8.0E-06
2820203	32.1	male	61	p.Gln307Ter	stop_gained	2.5E-06	46	.	8.0E-06	7.0E-06	8.0E-06
1018788	30.1	female	64	p.Glu308Lys	missense_variant	1.5E-05	29.4	0.64	8.0E-06	1.4E-05	8.0E-06
1692166	31.3	male	66	p.Glu308Lys	missense_variant	1.5E-05	29.4	0.64	8.0E-06	1.4E-05	8.0E-06
2057577	24.2	female	61	p.Glu308Lys	missense_variant	1.5E-05	29.4	0.64	8.0E-06	1.4E-05	8.0E-06
2218291	21.9	female	59	p.Glu308Lys	missense_variant	1.5E-05	29.4	0.64	8.0E-06	1.4E-05	8.0E-06
4499023	31.0	female	63	p.Glu308Lys	missense_variant	1.5E-05	29.4	0.64	8.0E-06	1.4E-05	8.0E-06
4706163	26.1	male	45	p.Glu308Lys	missense_variant	1.5E-05	29.4	0.64	8.0E-06	1.4E-05	8.0E-06
2622690	31.6	female	54	p.Glu308Val	missense_variant	2.5E-06	29.5	0.77	5.6E-05	3.5E-05	8.0E-06
3471612	26.7	male	57	p.Ile316Ser	missense_variant	7.5E-06	34	0.67	.	1.4E-05	2.0E-05
5110819	33.8	female	60	p.Ile316Ser	missense_variant	7.5E-06	34	0.67	.	1.4E-05	2.0E-05
5624110	27.5	female	66	p.Ile316Ser	missense_variant	7.5E-06	34	0.67	.	1.4E-05	2.0E-05
4713760	43.1	female	53	p.Leu322TrpfsTer33	frameshift_variant	2.5E-06	.	.	.	.	.

2014144	41.4	female	65	p.Leu325Phe	missense_variant	2.5E-06	15.6	0.39	.	.	.
2207685	29.6	male	70	p.Tyr332Cys	missense_variant	1.7E-05	26.8	0.36	2.4E-05	2.8E-05	.
2488699	21.7	female	65	p.Tyr332Cys	missense_variant	1.7E-05	26.8	0.36	2.4E-05	2.8E-05	.
3593904	26.0	male	54	p.Tyr332Cys	missense_variant	1.7E-05	26.8	0.36	2.4E-05	2.8E-05	.
4392500	30.3	male	60	p.Tyr332Cys	missense_variant	1.7E-05	26.8	0.36	2.4E-05	2.8E-05	.
4848184	20.9	female	54	p.Tyr332Cys	missense_variant	1.7E-05	26.8	0.36	2.4E-05	2.8E-05	.
5303549	26.2	male	42	p.Tyr332Cys	missense_variant	1.7E-05	26.8	0.36	2.4E-05	2.8E-05	.
5912744	25.4	female	59	p.Tyr332Cys	missense_variant	1.7E-05	26.8	0.36	2.4E-05	2.8E-05	.
1233912	27.2	male	50	p.Tyr332His	missense_variant	7.5E-06	24.7	0.38	1.6E-05	7.0E-06	8.0E-06
2539121	24.8	female	46	p.Tyr332His	missense_variant	7.5E-06	24.7	0.38	1.6E-05	7.0E-06	8.0E-06
4633126	22.9	female	58	p.Tyr332His	missense_variant	7.5E-06	24.7	0.38	1.6E-05	7.0E-06	8.0E-06
1092752	36.4	female	55	p.Arg18Leu	missense_variant	3.5E-05	20.7	0.31	4.0E-05	4.2E-05	1.2E-05
1117247	36.4	male	50	p.Arg18Leu	missense_variant	3.5E-05	20.7	0.31	4.0E-05	4.2E-05	1.2E-05
1383182	29.1	female	41	p.Arg18Leu	missense_variant	3.5E-05	20.7	0.31	4.0E-05	4.2E-05	1.2E-05
1727279	28.0	female	45	p.Arg18Leu	missense_variant	3.5E-05	20.7	0.31	4.0E-05	4.2E-05	1.2E-05
1804807	26.2	male	49	p.Arg18Leu	missense_variant	3.5E-05	20.7	0.31	4.0E-05	4.2E-05	1.2E-05
1814496	24.8	female	54	p.Arg18Leu	missense_variant	3.5E-05	20.7	0.31	4.0E-05	4.2E-05	1.2E-05
2254745	25.3	female	55	p.Arg18Leu	missense_variant	3.5E-05	20.7	0.31	4.0E-05	4.2E-05	1.2E-05
3840021	34.7	male	49	p.Arg18Leu	missense_variant	3.5E-05	20.7	0.31	4.0E-05	4.2E-05	1.2E-05
4057465	30.0	male	50	p.Arg18Leu	missense_variant	3.5E-05	20.7	0.31	4.0E-05	4.2E-05	1.2E-05
4269020	25.6	male	62	p.Arg18Leu	missense_variant	3.5E-05	20.7	0.31	4.0E-05	4.2E-05	1.2E-05
4276981	26.6	male	49	p.Arg18Leu	missense_variant	3.5E-05	20.7	0.31	4.0E-05	4.2E-05	1.2E-05
4924573	28.4	male	67	p.Arg18Leu	missense_variant	3.5E-05	20.7	0.31	4.0E-05	4.2E-05	1.2E-05
5602800	24.5	NA	43	p.Arg18Leu	missense_variant	3.5E-05	20.7	0.31	4.0E-05	4.2E-05	1.2E-05
5633683	34.1	female	56	p.Arg18Leu	missense_variant	3.5E-05	20.7	0.31	4.0E-05	4.2E-05	1.2E-05
3122152	29.1	male	52	p.Ile63Thr	missense_variant	2.5E-06	26.4	0.28	.	.	.
2657185	22.8	female	59	p.Lys73Asn	missense_variant	2.5E-06	26	0.28	.	.	.
4440479	30.5	female	62	p.Ala89Ser	missense_variant	2.5E-06	23.5	0.33	.	.	.

4526715	36.8	female	52	p.Thr203Ile	missense_variant	2.5E-06	23.6	0.27	.	.	.
1443337	28.1	female	50	p.Ala227Thr	missense_variant	1.3E-05	24.1	0.26	8.0E-06	7.0E-06	8.0E-06
2803033	24.1	female	52	p.Ala227Thr	missense_variant	1.3E-05	24.1	0.26	8.0E-06	7.0E-06	8.0E-06
4278945	25.6	female	58	p.Ala227Thr	missense_variant	1.3E-05	24.1	0.26	8.0E-06	7.0E-06	8.0E-06
5198492	29.8	female	48	p.Ala227Thr	missense_variant	1.3E-05	24.1	0.26	8.0E-06	7.0E-06	8.0E-06
5606745	25.4	female	61	p.Ala227Thr	missense_variant	1.3E-05	24.1	0.26	8.0E-06	7.0E-06	8.0E-06
1056788	NA	NA	NA	p.Gly231Ser	missense_variant	1.0E-05	22.3	0.25	3.2E-05	2.8E-05	6.0E-05
1389940	30.1	male	50	p.Gly231Ser	missense_variant	1.0E-05	22.3	0.25	3.2E-05	2.8E-05	6.0E-05
2626774	28.9	female	58	p.Gly231Ser	missense_variant	1.0E-05	22.3	0.25	3.2E-05	2.8E-05	6.0E-05
5817246	26.8	male	49	p.Gly231Ser	missense_variant	1.0E-05	22.3	0.25	3.2E-05	2.8E-05	6.0E-05
1231130	25.6	female	67	p.Arg236Cys	missense_variant	9.7E-05	25	0.29	4.8E-05	4.9E-05	7.2E-05
1358347	28.5	female	65	p.Arg236Cys	missense_variant	9.7E-05	25	0.29	4.8E-05	4.9E-05	7.2E-05
1658266	23.7	female	55	p.Arg236Cys	missense_variant	9.7E-05	25	0.29	4.8E-05	4.9E-05	7.2E-05
1895633	28.6	female	60	p.Arg236Cys	missense_variant	9.7E-05	25	0.29	4.8E-05	4.9E-05	7.2E-05
1897443	29.0	male	49	p.Arg236Cys	missense_variant	9.7E-05	25	0.29	4.8E-05	4.9E-05	7.2E-05
2001523	23.4	female	53	p.Arg236Cys	missense_variant	9.7E-05	25	0.29	4.8E-05	4.9E-05	7.2E-05
2019732	NA	NA	NA	p.Arg236Cys	missense_variant	9.7E-05	25	0.29	4.8E-05	4.9E-05	7.2E-05
2123437	31.8	female	48	p.Arg236Cys	missense_variant	9.7E-05	25	0.29	4.8E-05	4.9E-05	7.2E-05
2265984	24.6	male	59	p.Arg236Cys	missense_variant	9.7E-05	25	0.29	4.8E-05	4.9E-05	7.2E-05
2344149	25.7	male	58	p.Arg236Cys	missense_variant	9.7E-05	25	0.29	4.8E-05	4.9E-05	7.2E-05
2377099	33.8	female	66	p.Arg236Cys	missense_variant	9.7E-05	25	0.29	4.8E-05	4.9E-05	7.2E-05
2378871	22.9	male	51	p.Arg236Cys	missense_variant	9.7E-05	25	0.29	4.8E-05	4.9E-05	7.2E-05
2691597	22.4	male	62	p.Arg236Cys	missense_variant	9.7E-05	25	0.29	4.8E-05	4.9E-05	7.2E-05
2747825	22.8	male	64	p.Arg236Cys	missense_variant	9.7E-05	25	0.29	4.8E-05	4.9E-05	7.2E-05
2959889	25.5	male	52	p.Arg236Cys	missense_variant	9.7E-05	25	0.29	4.8E-05	4.9E-05	7.2E-05
3166667	26.8	female	61	p.Arg236Cys	missense_variant	9.7E-05	25	0.29	4.8E-05	4.9E-05	7.2E-05
3365275	26.5	male	63	p.Arg236Cys	missense_variant	9.7E-05	25	0.29	4.8E-05	4.9E-05	7.2E-05
3426635	22.9	female	51	p.Arg236Cys	missense_variant	9.7E-05	25	0.29	4.8E-05	4.9E-05	7.2E-05

3730837	30.9	female	41	p.Arg236Cys	missense_variant	9.7E-05	25	0.29	4.8E-05	4.9E-05	7.2E-05
3783463	24.0	female	49	p.Arg236Cys	missense_variant	9.7E-05	25	0.29	4.8E-05	4.9E-05	7.2E-05
3951669	24.7	female	56	p.Arg236Cys	missense_variant	9.7E-05	25	0.29	4.8E-05	4.9E-05	7.2E-05
3995742	27.5	female	57	p.Arg236Cys	missense_variant	9.7E-05	25	0.29	4.8E-05	4.9E-05	7.2E-05
4057045	29.3	male	55	p.Arg236Cys	missense_variant	9.7E-05	25	0.29	4.8E-05	4.9E-05	7.2E-05
4087507	26.0	female	58	p.Arg236Cys	missense_variant	9.7E-05	25	0.29	4.8E-05	4.9E-05	7.2E-05
4287970	26.5	female	59	p.Arg236Cys	missense_variant	9.7E-05	25	0.29	4.8E-05	4.9E-05	7.2E-05
4289858	19.8	female	56	p.Arg236Cys	missense_variant	9.7E-05	25	0.29	4.8E-05	4.9E-05	7.2E-05
4315127	19.7	female	65	p.Arg236Cys	missense_variant	9.7E-05	25	0.29	4.8E-05	4.9E-05	7.2E-05
4358102	20.8	male	56	p.Arg236Cys	missense_variant	9.7E-05	25	0.29	4.8E-05	4.9E-05	7.2E-05
4422258	25.2	female	54	p.Arg236Cys	missense_variant	9.7E-05	25	0.29	4.8E-05	4.9E-05	7.2E-05
4508531	NA	NA	NA	p.Arg236Cys	missense_variant	9.7E-05	25	0.29	4.8E-05	4.9E-05	7.2E-05
4984447	36.8	female	60	p.Arg236Cys	missense_variant	9.7E-05	25	0.29	4.8E-05	4.9E-05	7.2E-05
5038633	27.1	male	45	p.Arg236Cys	missense_variant	9.7E-05	25	0.29	4.8E-05	4.9E-05	7.2E-05
5073258	26.9	female	45	p.Arg236Cys	missense_variant	9.7E-05	25	0.29	4.8E-05	4.9E-05	7.2E-05
5102117	35.0	female	66	p.Arg236Cys	missense_variant	9.7E-05	25	0.29	4.8E-05	4.9E-05	7.2E-05
5150940	33.3	female	61	p.Arg236Cys	missense_variant	9.7E-05	25	0.29	4.8E-05	4.9E-05	7.2E-05
5326327	46.3	female	53	p.Arg236Cys	missense_variant	9.7E-05	25	0.29	4.8E-05	4.9E-05	7.2E-05
5698952	29.6	female	59	p.Arg236Cys	missense_variant	9.7E-05	25	0.29	4.8E-05	4.9E-05	7.2E-05
5705636	30.5	female	61	p.Arg236Cys	missense_variant	9.7E-05	25	0.29	4.8E-05	4.9E-05	7.2E-05
5978324	23.8	male	68	p.Arg236Cys	missense_variant	9.7E-05	25	0.29	4.8E-05	4.9E-05	7.2E-05
2640076	26.0	male	41	p.Arg236Gly	missense_variant	2.5E-06	26.3	0.26	.	.	.
5792270	20.7	female	56	p.Met281Ile	missense_variant	2.5E-06	23.4	0.28	8.0E-06	.	.
1197045	25.2	female	64	p.Gly323Val	missense_variant	2.5E-06	20.7	0.25	.	1.4E-05	1.2E-05

Supplementary table 2

Deleterious variants in BSN in UKBB cohort

proband	Sex	Age-recruitment	European	BMI	HGVSP	CADD_phred	gnomAD3_AF
3376698	female	49	NA	24.2	Q211+2T>G	24.8	.
1298264	male	60	Y	41.2	F498S	32	.
5736922	female	62	Y	27.2	W510C	33	.
3215658	female	58	Y	36.0	K571X	35	.
3951330	female	61	Y	NA	E662+1G>A	23.6	.
1004191	male	60	Y	28.3	E662+2T>A	22.6	6.99E-06
2802610	male	51	Y	38.8	E662+2T>A	22.6	6.99E-06
2059217	male	45	Y	29.7	T714fs	26.5	.
1372818	male	41	Y	45.5	G1253fs	19.59	.
5004856	male	50	Y	29.1	R1276X	35	.
2311136	male	44	Y	31.6	R1276X	35	.
3531133	female	65	Y	31.7	F1323fs	29.4	.
2381858	female	66	Y	23.9	G1377fs	27.5	.
1517223	male	66	Y	36.8	R1447X	26.2	.
1367126	female	64	Y	46.8	G1494fs	21.8	.
5028981	female	51	Y	30.9	V1834fs	24.9	.
2561154	male	41	Y	22.7	V1834fs	24.9	.
5638004	female	62	Y	25.8	A1929fs	23.7	.
4492069	female	59	Y	45.7	Q1958fs	26.6	.
2992473	male	52	Y	30.8	Y2073X	27.7	.
3348582	female	55	Y	41.2	Q2334X	37	.
3424371	male	43	Y	31.8	Q2451X	38	.
5603259	male	61	Y	31.1	Q2464X	39	.
3472038	female	63	NA	25.2	R2486X	37	.
1059118	female	44	Y	42.6	L2846fs	33	.
5342528	male	63	Y	33.3	P2915fs	26.1	.
4911564	female	62	Y	32.3	Q3052X	38	.
4452519	female	47	Y	47.2	Y3284fs	35	.
2104485	male	65	NA	28.6	Q3541X	43	.
2714273	female	68	Y	33.1	Q3707X	42	.
5896339	female	64	Y	27.0	Q3707X	42	.
1677591	male	55	Y	35.3	Q3854fs	33	.

Supplementary table 3

UKBB phenome-wide association for BSN LGD variants (24 variants)

trait	BETA	SE	Pvalue
Whole_body_fat_mass	12.50	1.86	1.63E-11
Arm_fat_mass_(right)	0.81	0.12	2.14E-11
Arm_fat_mass_(left)	0.89	0.13	4.02E-11
Leg_fat_mass_(right)	1.90	0.31	5.76E-10
Leg_fat_mass_(left)	1.81	0.30	1.45E-09
Trunk_fat_mass	5.91	1.02	6.74E-09
Leg_fat-free_mass_(left)	1.35	0.24	2.06E-08
Leg_predicted_mass_(left)	1.26	0.23	2.47E-08
Leg_predicted_mass_(right)	1.21	0.23	8.90E-08
Leg_fat-free_mass_(right)	1.28	0.24	1.11E-07
Arm_fat_percentage_(right)	7.42	1.46	3.83E-07
Arm_fat_percentage_(left)	7.40	1.47	5.19E-07
Arm_fat-free_mass_(left)	0.46	0.09	7.44E-07
Arm_fat-free_mass_(right)	0.42	0.09	1.46E-06
Whole_body_water_mass	4.40	0.92	1.54E-06
Arm_predicted_mass_(right)	0.39	0.08	1.78E-06
Arm_predicted_mass_(left)	0.42	0.09	1.99E-06
Whole_body_fat-free_mass	5.78	1.24	3.19E-06
Body_fat_percentage	5.72	1.24	4.39E-06
Leg_fat_percentage_(right)	4.70	1.08	1.35E-05
Trunk_fat_percentage	6.09	1.43	1.97E-05
Leg_fat_percentage_(left)	4.20	1.03	4.39E-05
Trunk_fat-free_mass	2.28	0.64	3.61E-04
Trunk_predicted_mass	2.16	0.61	4.30E-04
High_light_scatter_reticulocyte_count	0.01	0.00	1.82E-03
High_light_scatter_reticulocyte_percentage	0.12	0.07	7.23E-02

Supplementary table 4

ICD10 classification enrichment test for 27 BSN carriers in the 145103 UKBB European cohort

ICD10	meaning	#Affected	#Affected BSN_carriers	p-value
Z824	Family history of ischaemic heart disease and other diseases of the circulatory system	6546	6	1.10E-03
I120	Hypertensive renal disease with renal failure	438	2	3.04E-03
I10	Essential (primary) hypertension	39608	14	7.90E-03
K768	Other specified diseases of liver	722	2	8.00E-03

Supplementary table 5

BSN pLoF variants in All of US dataset

**Index**	**Age**	**SEX**	**BMI**	**P. ancestry**	**HGVSc**	**HGVSp**	**VariantFunction**
1	46-50	F	33	EUR	c.594C>A	p.Cys198Ter	stop_gained
2	46-50	M	36	EUR	c.1990del	p.Leu664TrpfsTer50	frameshift_variant
3	46-50	F	43	EUR	c.3688C>T	p.Gln1230Ter	stop_gained
4	51-55	F	38	AFR	c.3826C>T	p.Arg1276Ter	stop_gained
5	51-55	F	38	EUR	c.5602G>T	p.Gly1868Ter	stop_gained
6	66-70	M	30	EUR	c.5873_5874del	p.Gln1958ArgfsTer9	frameshift_variant
7	66-70	M	22	AMR	c.7177G>T	p.Glu2393Ter	stop_gained
8	26-30	F	25	AFR	c.7390C>T	p.Gln2464Ter	stop_gained
9	61-65	F	35	EUR	c.7792_7793del	p.Leu2598GlufsTer11	frameshift_variant
10	56-60	F	34	EUR	c.8641-2A>G	p.Pro2881splice	splice_acceptor_variant
11	66-70	F	43	EUR	c.9184C>T	p.Gln3062Ter	stop_gained
12	36-40	M	27	AFR	c.9463C>T	p.Gln3155Ter	stop_gained
13	51-55	F	32	AMR	c.9988C>T	p.Arg3330Ter	stop_gained
14	66-70	F	45	AFR	c.11551_11552insGGGT	p.Ala3851GlyfsTer139	frameshift_variant
15	56-60	M	41	EUR	c.11509-5_11524del	p.Pro3887splice	splice_acceptor_variant
