# Supplementary material for: Rare predicted loss of function alleles in Bassoon (BSN) are associated with obesity
Source: NPJ Genom Med. 2023 Oct 21;8:33. doi: 10.1038/s41525-023-00376-7 (PMC10590409; doi:10.1038/s41525-023-00376-7)
Supplement: Supplementary file 2 — REPORTING SUMMARY [file 41525_2023_376_MOESM2_ESM.pdf]

nature portfolio

Corresponding author(s): Rudolph Leibel

Last updated by author(s): 2023-20-07

Reporting Summary

Nature Portfolio wishes to improve the reproducibility of the work that we publish. This form provides structure for consistency and transparency in reporting. For further information on Nature Portfolio policies, see our Editorial Policies and the Editorial Policy Checklist.

Statistics

For all statistical analyses, confirm that the following items are present in the figure legend, table legend, main text, or Methods section.

n/a	Confirmed	
☐	☒	The exact sample size (*n*) for each experimental group/condition, given as a discrete number and unit of measurement
☐	☒	A statement on whether measurements were taken from distinct samples or whether the same sample was measured repeatedly
☐	☒	The statistical test(s) used AND whether they are one- or two-sided<br>*Only common tests should be described solely by name; describe more complex techniques in the Methods section.*
☐	☒	A description of all covariates tested
☐	☒	A description of any assumptions or corrections, such as tests of normality and adjustment for multiple comparisons
☐	☒	A full description of the statistical parameters including central tendency (e.g. means) or other basic estimates (e.g. regression coefficient) AND variation (e.g. standard deviation) or associated estimates of uncertainty (e.g. confidence intervals)
☐	☒	For null hypothesis testing, the test statistic (e.g. *F*, *t*, *r*) with confidence intervals, effect sizes, degrees of freedom and *P* value noted<br>*Give P values as exact values whenever suitable.*
☐	☒	For Bayesian analysis, information on the choice of priors and Markov chain Monte Carlo settings
☐	☒	For hierarchical and complex designs, identification of the appropriate level for tests and full reporting of outcomes
☐	☒	Estimates of effect sizes (e.g. Cohen's *d*, Pearson's *r*), indicating how they were calculated

Our web collection on statistics for biologists contains articles on many of the points above.

Software and code

Policy information about availability of computer code

Data collection	No software was used to collect the data.
Data analysis	We used regenie for statistical analysis. The software is written by a team in Regeneron, available on GitHub: https://nam02.safelinks.protection.outlook.com/?url=https%3A%2F%2Frgcgithub.github.io%2Fregenie%2F&data=05%7C01%7Ccal8%40cumc.columbia.edu%7Cb05e1e0311ea4ad5915308db8868e3e7%7Cb0002a9b0017404d97dc3d3bab09be81%7C0%7C0%7C638253756300918647%7CUnknown%7CTWFpbGZsb3d8eyJWIjoiMC4wLjAwMDAiLCJQIjoiV2luMzIiLCJBTiI6Ik1haWwiLCJXVCI6Mn0%3D%7C3000%7C%7C%7C&sdata=bN1OqAtL1w6zrFQlNHvmmRAXt5KbQJd9zlTUAyN6jdg%3D&reserved=0

For manuscripts utilizing custom algorithms or software that are central to the research but not yet described in published literature, software must be made available to editors and reviewers. We strongly encourage code deposition in a community repository (e.g. GitHub). See the Nature Portfolio guidelines for submitting code & software for further information.

Data

Policy information about availability of data

All manuscripts must include a data availability statement. This statement should provide the following information, where applicable:

- Accession codes, unique identifiers, or web links for publicly available datasets
- A description of any restrictions on data availability
- For clinical datasets or third party data, please ensure that the statement adheres to our policy

Researchers can access All of Us data at All of Us Research Hub (researchallofus.org)
The UK Biobank data is availavle from https://www.ukbiobank.ac.uk/

Research involving human participants, their data, or biological material

Policy information about studies with human participants or human data. See also policy information about sex, gender (identity/presentation), and sexual orientation and race, ethnicity and racism.

Reporting on sex and gender	Reported sex was used as a covariate. Effect size is seen independent of sex.
Reporting on race, ethnicity, or other socially relevant groupings	We restricted samples to only samples that were found by PCA plot to be EUR in origin.
Population characteristics	Samples that were coded as having had cancer, eating disorders, or both were removed from the cohort prior to analysis. Samples were also removed for relatedness; relatedness was estimated using plink King, when sample pairs had a relatedness greater than 0.12 (second degree relative or closer) the sample that had more relatedness to the overall cohort was excluded
Recruitment	The Columbia cohort was recruited for families with extreme obesity.
Ethics oversight	All studies were under the auspices of the Columbia University IRB "Molecular Genetic Analysis of Obesity and Non-Insulin Dependent Diabetes Mellitus" IRB #: AAAA4485 which expires on 5/1/24.

Note that full information on the approval of the study protocol must also be provided in the manuscript.

Field-specific reporting

Please select the one below that is the best fit for your research. If you are not sure, read the appropriate sections before making your selection.

☒ Life sciences ☐ Behavioural & social sciences ☐ Ecological, evolutionary & environmental sciences

For a reference copy of the document with all sections, see nature.com/documents/nr-reporting-summary-flat.pdf

Life sciences study design

All studies must disclose on these points even when the disclosure is negative.

Sample size	UKBB had 167246 EUR samples at time of analysis, we used entire dataset
Data exclusions	Samples were excluded for prior cancer, eating disorder, or both. We also excluded closely related individuals. This was estimated using plink King, when sample pairs had a relatedness greater than 0.12 (second degree relative or closer) the sample that had more relatedness to the overall cohort was excluded
Replication	Confirmation cohort was done on the 48,722 EUR samples in the All of Us dataset.
Randomization	No randomization
Blinding	Analysis was done blinded.

Reporting for specific materials, systems and methods

We require information from authors about some types of materials, experimental systems and methods used in many studies. Here, indicate whether each material, system or method listed is relevant to your study. If you are not sure if a list item applies to your research, read the appropriate section before selecting a response.

Materials & experimental systems

n/a	Involved in the study
☒	☐ Antibodies
☒	☐ Eukaryotic cell lines
☒	☐ Palaeontology and archaeology
☒	☐ Animals and other organisms
☒	☐ Clinical data
☒	☐ Dual use research of concern
☒	☐ Plants

Methods

n/a	Involved in the study
☒	☐ ChIP-seq
☒	☐ Flow cytometry
☒	☐ MRI-based neuroimaging
